# Carcinoembryonic Antigen-Related Cell Adhesion Molecule Type 5 Receptor-Targeted Fluorescent Intraoperative Molecular Imaging Tracer for Lung Cancer: A Nonrandomized Controlled Trial

**DOI:** 10.1001/jamanetworkopen.2022.52885

**Published:** 2023-01-03

**Authors:** Feredun Azari, Ruben P. J. Meijer, Gregory T. Kennedy, Andrew Hanna, Ashley Chang, Bilal Nadeem, Azra Din, André Pèlegrin, Bérénice Framery, Françoise Cailler, Neil T. Sullivan, John Kucharczuk, Linda W. Martin, Alexander L. Vahrmeijer, Sunil Singhal

**Affiliations:** Department of Thoracic Surgery, University of Pennsylvania, Philadelphia; Centre for Human Drug Research, Department of Surgery, Leiden University Medical Center, Leiden, the Netherlands; Department of Thoracic Surgery, University of Pennsylvania, Philadelphia; Department of Surgery, University of Pennsylvania Perelman School of Medicine, Philadelphia; Department of Thoracic Surgery, University of Pennsylvania, Philadelphia; Department of Thoracic Surgery, University of Pennsylvania, Philadelphia; Department of Thoracic Surgery, University of Pennsylvania, Philadelphia; Institute of Cancer Research of Montpellier, University of Montpellier, Montpellier, France, SurgiMab, Montpellier, France; SurgiMab, Montpellier, France; SurgiMab, Montpellier, France; Department of Thoracic Surgery, University of Pennsylvania, Philadelphia; Department of Thoracic Surgery, University of Pennsylvania, Philadelphia; Department of Thoracic Surgery, University of Virginia School of Medicine, Charlottesville; Centre for Human Drug Research, Department of Surgery, Leiden University Medical Center, Leiden, the Netherlands; Department of Thoracic Surgery, University of Pennsylvania, Philadelphia

## Abstract

**IMPORTANCE:**

Localization of subcentimeter ground glass opacities during minimally invasive thoracoscopic lung cancer resections is a significant challenge in thoracic oncology. Intraoperative molecular imaging has emerged as a potential solution, but the availability of suitable fluorescence agents is a limiting factor.

**OBJECTIVE:**

To evaluate the suitability of SGM-101, a carcinoembryonic antigen–related cell adhesion molecule type 5 (CEACAM5) receptor–targeted near-infrared fluorochrome, for molecular imaging–guided lung cancer resections, because glycoprotein is expressed in more than 80% of adenocarcinomas.

**DESIGN, SETTING, AND PARTICIPANTS:**

For this nonrandomized, proof-of-principal, phase 1 controlled trial, patients were divided into 2 groups between August 1, 2020, and January 31, 2022. Patients with known CEACAM5-positive gastrointestinal tumors suggestive of lung metastasis were selected as proof-of-principle positive controls. The investigative group included patients with lung nodules suggestive of primary lung malignant neoplasms. Patients 18 years or older without significant comorbidities that precluded surgical exploration with suspicious pulmonary nodules requiring surgical biopsy were included in the study.

**INTERVENTIONS:**

SGM-101 (10 mg) was infused up to 5 days before index operation, and pulmonary nodules were imaged using a near-infrared camera system with a dedicated thoracoscope.

**MAIN OUTCOMES AND MEASURES:**

SGM-101 localization to pulmonary nodules and its correlation with CEACAM5 glycoprotein expression by the tumor as quantified by tumor and normal pulmonary parenchymal fluorescence.

**RESULTS:**

Ten patients (5 per group; 5 male and 5 female; median [IQR] age, 66 [58–69] years) with 14 total lesions (median [range] lesion size, 0.91 [0.90–2.00] cm) were enrolled in the study. In the control group of 4 patients (1 patient did not undergo surgical resection because of abnormal preoperative cardiac clearance findings that were not deemed related to SGM-101 infusion), the mean (SD) lesion size was 1.33 (0.48) cm, 2 patients had elevated serum CEA markers, and 2 patients had normal serum CEA levels. Of the 4 patients who underwent surgical intervention, those with 2+ and 3+ tissue CEACAM5 expression had excellent tumor fluorescence, with a mean (SD) tumor to background ratio of 3.11 (0.45). In the patient cohort, the mean (SD) lesion size was 0.68 (0.22) cm, and no elevations in serum CEA levels were found. Lack of SGM-101 fluorescence was associated with benign lesions and with lack of CEACAM5 staining.

**CONCLUSIONS AND RELEVANCE:**

This in-human proof-of-principle nonrandomized controlled trial demonstrated SGM-101 localization to CEACAM5-positive tumors with the detection of real-time near-infrared fluorescence in situ, ex vivo, and by immunofluorescence microscopy. These findings suggest that SGM-101 is a safe, receptor-specific, and feasible intraoperative molecular imaging fluorochrome that should be further evaluated in randomized clinical trials.

## Introduction

Lung cancer, particularly non–small cell lung cancer (NSCLC), carries one of the highest rates of cancer-related morbidity and mortality worldwide.^1^ Although implementation of screening programs in high-risk patient groups and education around tobacco use prevention have made an impact over the past few decades, NSCLC still inflicts significant strains on the general population.^2^ Despite the introduction of advanced immune checkpoint treatments and targeted radiotherapy regimens, surgery remains the best tool in the armamentarium against the disease.^3,4^ Stage by stage, surgery confers the highest survival probability in nondisseminated disease.^5^ However, success of surgical intervention is intimately related to oncologically sound disease removal by ensuring negative margins and identification of occult synchronous or metachronous lesions. Recurrence after surgery is correlated with inferior survival outcomes.^6^

Therefore, thoracic surgeons continually strive for R0 oncologic disease clearance at index operation.^7^ Surgeons have traditionally relied on sensory feedback, such as visualization and tactile assessment, to confirm lesion localization and removal. However, with the increasing use of minimally invasive techniques, these core techniques have been continually challenged, as the surgeon has to rely on the visual field achieved via a thoracoscope or assess lesion characteristics ex vivo on the back table.^7^ Intraoperative molecular imaging (IMI) has recently emerged as a potential solution to circumvent the current challenges.^7–9^ This technology involves systemic delivery of tumor-targeted fluorochrome, which is then visualized using specialized near-infrared (NIR) (700–2526 nm) camera systems.^7^ Several types of fluorochromes have been approved for a variety of clinical uses by the US Food and Drug Administration (FDA). These fluorochromes include indocyanine green, pafolacianine, and gleolan, which improve outcomes in various solid organ malignant tumors, including sarcomas, lung cancer, colorectal cancer, breast cancer, intracranial tumors, and ovarian cancer. However, due to the large heterogeneity that exists within various NSCLC tumors, particularly lung adenocarcinomas, not all patients benefit from currently available NIR tracers.^8,10–15^

A promising target is the glycoprotein carcinoembryonic antigen–related cell adhesion molecule type 5 (CEACAM5), also known as carcinoembryonic antigen (CEA). Although the glycoprotein has been extensively studied and used for screening, diagnosis, and prognosis in gastrointestinal malignant neoplasms, it has been relatively underused in lung cancer, although 30% to 80% of lung adenocarcinomas express this surface receptor.^16–20^ In addition, the surface glycoprotein expression in CEACAM5-positive tumors is significantly higher than that in normal tissue, making it an attractive antigen to be explored (10^6^ surface antigens per cell).^21–23^ Targeted delivery of anti-CEACAM5–conjugated NIR fluorochromes, such as SGM-101 (Surgimab), has been demonstrated in colorectal and pancreatic adenocarcinomas, with encouraging results.^21,24–26^ SGM-101 is an anti-CEACAM5 antibody conjugated to the NIR BM-104 fluorochrome that specifically emits fluorescence once it binds to CEACAM5-expressing tumors. Given the specificity of the fluorochrome, the ability to readily determine CEACAM5 tumor expression from preoperative biopsies, and the serum detection of glycoprotein, SGM-101 presents an intriguing area to explore in IMI-guided lung cancer resections.

In this study, we explored the safety, efficacy, and specificity of SGM-101 during IMI-guided lung nodule resections. First, we explored a large national cancer database for CEACAM5 RNA expression levels in various lung cancers. Second, we assessed glycoprotein expression levels in 33 patients diagnosed with lung adenocarcinoma from our institution to assess local rates of CEACAM5 tumor presence. Third, we performed an open-label nonrandomized controlled trial in patients with gastrointestinal tumors who presented with lung metastases based on serum CEA levels as a control and compared the results in subsequent patients who underwent SGM-101–guided primary lung cancer resection. We hypothesized that SGM-101 would specifically localize to CEACAM5-positive lung nodules and allow lesion localization in vivo, ex vivo, and on immunohistopathologic assessment.

## Methods

### Study Design and Patient Selection

The current study was an open-label, nonblinded, proof-of-principle, phase 1 nonrandomized controlled trial performed in patients 18 years or older at the Hospital of the University of Pennsylvania in Philadelphia from August 1, 2020, to January 31, 2022. The trial protocol appears in Supplement 1. Details on immunohistochemistry, The Cancer Genome Atlas analysis, inclusion and exclusion criteria, fluorochrome parameters, camera systems used, and outcomes analyzed are detailed in the eMethods in Supplement 2. Data on race and ethnicity were not collected because this was a cellular molecular study and such data would not be informative. The study was reviewed and approved by the University of Pennsylvania Institutional Review Board in accordance with Good Clinical Practice guidelines as outlined by the International Council of Harmonization of Technical Requirements for Pharmaceuticals for Human Use in addition to laws and regulations for human research by the state of Pennsylvania and the FDA. All patients were informed of the risks, benefits, and alternatives regarding the interventions in the study. Participants enrolled in the study signed written informed consent forms approved by the University of Pennsylvania Institutional Review Board before initiation of the trial. The rationale, background, methods, design, analysis, and interpretation of the study follow the recommendations set by the Transparent Reporting of Evaluations With Nonrandomized Designs (TREND) reporting guideline.

### Study Groups

In this nonrandomized controlled trial, the first group (positive control) included patients with known primary or recurrent gastrointestinal adenocarcinoma (colon, rectal, and pancreatic adenocarcinoma) with or without elevated serum CEA levels who were found to have nodules suggestive of pulmonary metastases. The second group included patients with nodules suggestive of malignant primary pulmonary disease on screening imaging who were scheduled for surgical resection regardless of serum CEA status. Of note, our institutional practice does not include preoperative nodule sampling.

### NIR Tracer

SGM-101 was manufactured in accordance with Good Laboratory Practice guidelines and supplied by Surgimab. The SGM-101 active ingredient is a covalent conjugate of the SGM-Ch511 anti-CEACAM5 chimeric monoclonal antibody with the fluorochrome BM-104. Patients in this study were administered 10 mg of SGM-101 intravenously for 30 minutes followed by a 50-mL flush of isotonic saline to account for the dead volume of the tubing. Patients were administered SGM-101 3 to 5 days (±1 day) before surgery at the Center for Health and Precision Surgery and monitored for a minimum of 1-hour after infusion.

### Surgical Procedures

Surgical procedures were performed by a thoracic surgeon (J.K. and S.S.). During surgery, surgeons used standard visualization and finger palpation (when applicable) to identify known tumors. After identification of the tumor, NIR imaging was used to confirm lesion fluorescence. If the preoperatively identified nodule was unidentifiable by white-light visualization or palpation, localization using fluorescence guidance was attempted. After the primary lesions were identified, fluorescence imaging was used to assess the hemithorax for occult lesions. After resection of the nodules, the specimen was analyzed ex vivo on a back table using an NIR camera system to assess nodule fluorescence and margin assessment. All samples were analyzed in triplicate by Pearl and Odyssey NIR Imager (LI-COR Biosciences) as well as ImageJ, version 1.53t (National Institutes of Health). The sample was then sent for frozen section analysis by a board-certified thoracic pathologist.

### Statistical Analysis

Data are presented as mean (SD) unless otherwise noted. Data were analyzed for parametric distribution. An unpaired *t* test was used to compare continuous variables across 2 groups, whereas analysis of variance was used to compare continuous variables across more than 2 groups. Univariate statistical significance between groups were confirmed using the Fisher exact test. A 1-sided *P* < .05 was considered statistically significant.

## Results

### Demographic Characteristics

A total of 35 patients were eligible for enrollment in the study, of whom 10 (5 per group; 5 male and 5 female; median [IQR] age, 66 [58–69] years) with 14 total lesions (median [range] lesion size, 0.91 [0.90–2.00] cm) consented to undergo a proof-of-principle SGM-101–guided lung resection clinical trial (eTables 1 and 2 in Supplement 2). There was no loss of follow-up in the study. Given that SGM-101 was successful in the localization of pancreatic and colorectal malignant neoplasms, we selected 5 patients with malignant neoplasms suggestive of colorectal or pancreatic cancer as a control group in the study before use of the tracer in the primary lung cancer group (eTable 2 in Supplement 2).^21,26^ After selection of the control group, we selected 5 patients for the primary lung malignant neoplasm cohort. All patients received 10 mg of SGM-101, with a median (range) time from infusion to surgery of 92.88 (91.2–94.67) hours. The median (range) preoperative serum CEA level was 3.0 ng/mL (2.0–3.5 ng/mL) (to convert to micrograms per liter, multiply by 1), with the metastasis cohort having an elevated median (range) CEA level of 5.11 ng/mL (3.10–9.18 ng/mL) (*P* = .03).

No SGM-101 infusion–related complications and no severe (Clavien-Dindo classification higher than 3) postoperative complications occurred. Patient 5 in the metastasis control cohort underwent infusion of SGM-101 but had abnormal electrocardiographic findings during preoperative clearance and did not undergo surgery. Demographic details and complication profiles are provided in eTables 2 and 3 in Supplement 2.

### Localization of CEACAM5-Positive Metastatic Nodules

**Figure 1.**
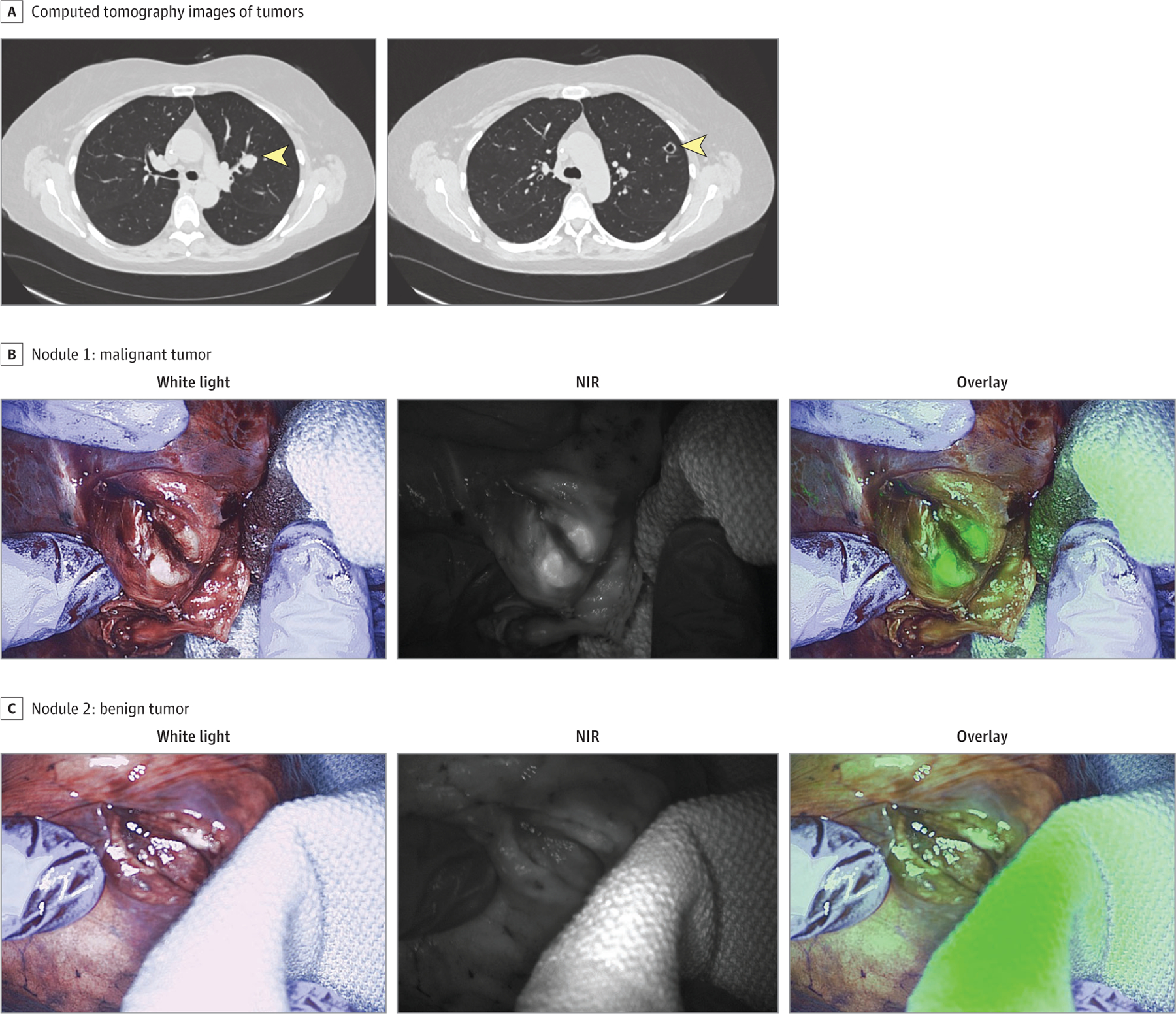
SGM-101–Guided Lung Nodule Resection in a Control Patient A, Computed tomography images of the tumor (yellow arrowheads indicate tumor). B, Nodule 1. C, Nodule 2. NIR indicates near infrared.

**Figure 2.**
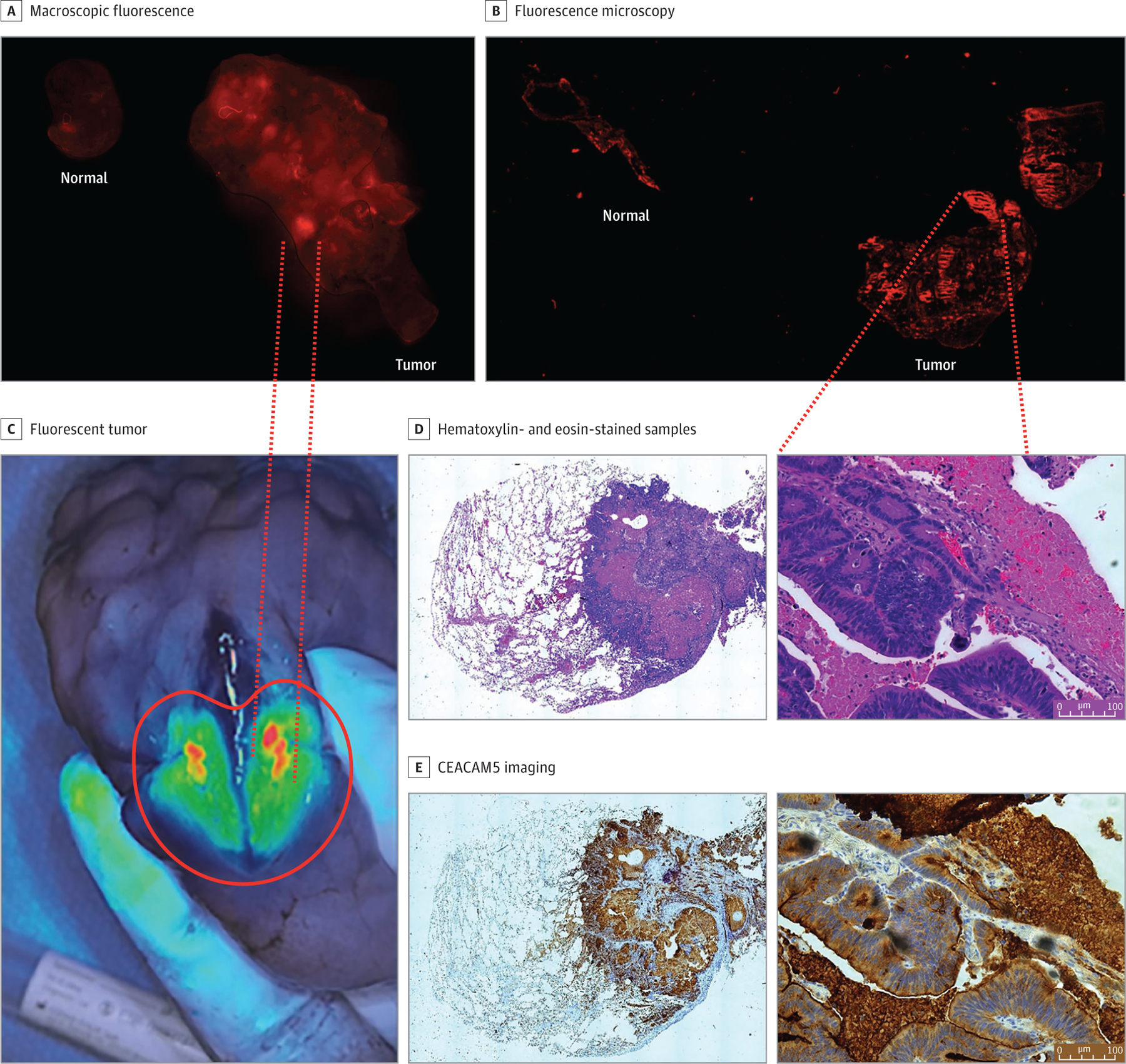
Association of SGM-101 Fluorescence With Carcinoembryonic Antigen–Related Cell Adhesion Molecule Type 5 (CEACAM5) Presence by Immunohistochemical Analysis Scale bars = 100 μm.

A total of 5 patients were recruited for the control group. One patient (patient 5) did not undergo surgical resection because of abnormal preoperative cardiac clearance findings that were not deemed related to SGM-101 infusion. The mean (SD) lesion size in the control cohort was 1.33 (0.48) cm. Patients 1 and 2 had elevated serum CEA markers, whereas patients 3 and 4 had normal serum CEA levels. Of the 4 patients who underwent surgical intervention, those with 2+ and 3+ tissue CEACAM5 expression had excellent tumor fluorescence, with a mean (SD) tumor to background ratio (TBR) of 3.11 (0.45) (Figure 1 and Figure 2; eFigures 1 and 2 in Supplement 2).

**Figure 3.**
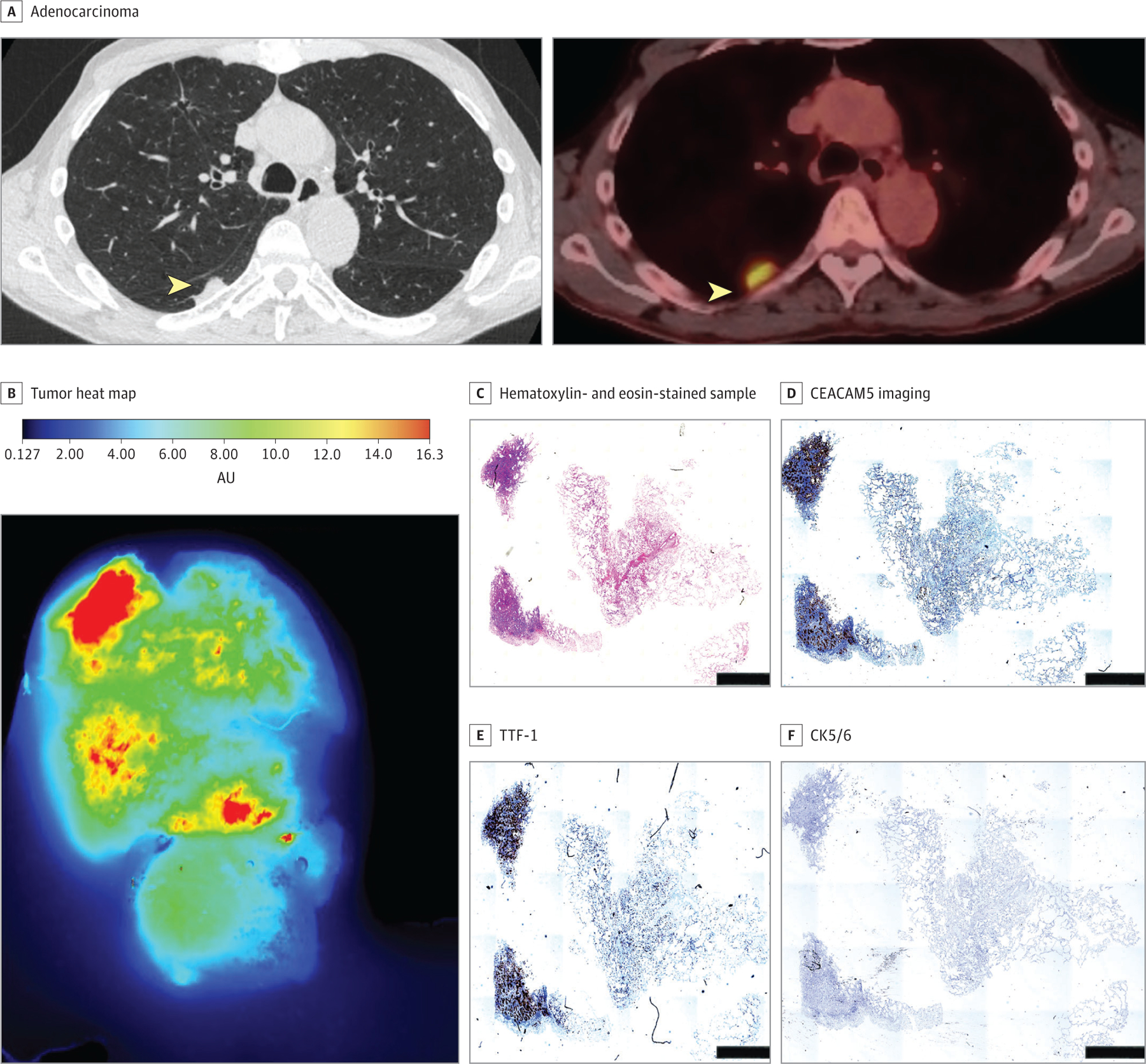
Localization of SGM-101 to Carcinoembryonic Antigen–Related Cell Adhesion Molecule Type 5 (CEACAM5) Tumors Patient 8 with invasive adenocarcinoma demonstrated a tumor with a large necrotic core (>80%) but had high CEACAM5 staining correlating with the thyroid transcription factor 1 (TTF-1) score (scale bar = 100 μm). Tumors were able to localize in vivo, ex vivo, and on a near-infrared scanner. AU indicates absorbance units; CK5/6, cytokeratin 5/6.

Patient 1 had 2 concerning nodules on preoperative workup (Figure 1 and Figure 2; eFigures 1, 3, and4 in Supplement 2). SGM-101 identified 1 nodule in vivo and ex vivo by fluorescence microscopy (Video). The mean (SD) TBR for nodule 1 was 3.12 (0.38). Nodule 2 was visualized in vivo but did not demonstrate any fluorescence in vivo or ex vivo (Video). The CEACAM5 staining demonstrated a 3+ score for nodule 1, and no staining was observed for nodule 2. Nodule 1 was confirmed to be a metastatic lesion associated with primary pathology (Figure 2 and Figure 3), whereas nodule 2 was a lymphoid aggregate. Similar observations were noted in patient 2, whereas serum CEA levels were elevated and demonstrated excellent fluorescence in vivo, ex vivo, and on histologic assessment (Video). The mean (SD) TBR was 2.89 (0.51) for the fluorescent nodule for patient 2. SGM-101 fluorescence correlated with 3+ CEACAM5 staining (Video; Figure 2; eFigures 2, 4, and 5 in Supplement 2).

Patient 3 had known stage 3 (T2N3M0) pancreatic adenocarcinoma that was operated on 6 months earlier. This patient had no serum CEA elevation. The nodule was not visualized in vivo but demonstrated mild CEACAM5 (1+) staining with fluorescence that was detected on ex vivo assessment. Retrospective analysis of the index operation revealed a normal serum CEA level at the time of pancreaticoduodenectomy with mild CEACAM5 staining on uncinate tumors. Conversely, in patient 4 with colorectal adenocarcinoma, who never had serum CEA elevation but had strong CEACAM5 staining at the time of index operation, nodules suggestive of disease were visualized in vivo and ex vivo and were associated with CEACAM5 staining (2+). Compared with patients 1 and 2, the nodule was significantly smaller (0.51 cm) and produced visible but statistically significantly lower fluorescence emission (mean [SD] TBR, 1.89 [0.33]; *P* = .04) (eFigure 5 in Supplement 2).

### SGM-101 Fluorescent Labeling of CEACAM5-Positive Lung Tumors

**Figure 4.**
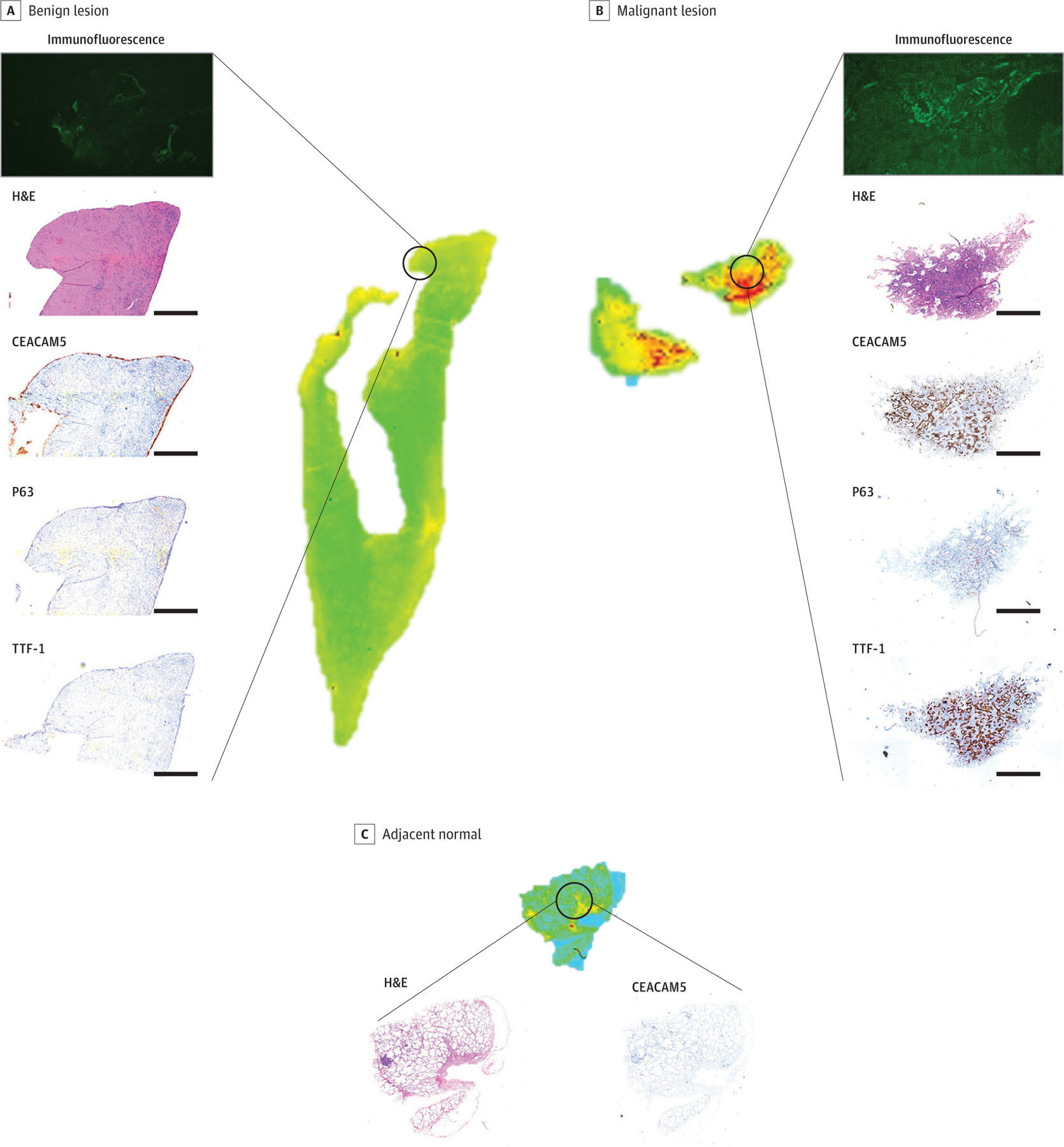
Fluorescence Microscopy Assessment of Primary Lung Nodules Analyzed in the Study The benign lesion (A) demonstrates no uptake of SGM-101 fluorescence, which corresponded with a lack of carcinoembryonic antigen, p63, and thyroid transcription factor 1 (TTF-1) staining (scale bar = 100 μm). Conversely, malignant lesions (primary lung adenocarcinoma) (B) demonstrate areas of fluorescence corresponding to areas of carcinoembryonic antigen–related cell adhesion molecule type 5 (CEACAM5) staining that were also congruent with areas of TTF-1 expression, demonstrating CEACAM5-dependent SGM-101 labeling. Adjacent normal lung parenchyma (inset, C) demonstrates a lack of fluorescence and a lack of CEACAM5 staining. H&E indicates hematoxylin-eosin.

Five patients with primary nodules were selected for a proof-of-principle study. No elevation in serum CEA levels were found in this patient cohort. The mean (SD) lesion size was 0.68 (0.22) cm. Lack of SGM-101 fluorescence was associated with benign lesions and with lack of CEACAM5 staining. Benign lesions lacked any fluorescence in ex vivo and histologic analyses (Figure 4). Patient 1 demonstrated an abscess cavity that was positive on positron emission tomography at preoperative assessment. No SGM-101 fluorescence, no CEACAM5 staining, and no other immunohistochemical staining were associated with markers of lung malignant neoplasms, including thyroid transcription factor 1 (TTF-1), cytokeratin 5/6 (CK 5/6), and p63 (Figure 4; eFigure 6 in Supplement 2). The presence of CEACAM5, particularly strong (>2+), was associated with the detection of SGM-101 fluorescence in vivo (Figure 4; eTables 4–6 in Supplement 2).

The area of fluorescence in patient 8 was lower than the lesion size, but on inspection, the tumor had a significant area of necrosis (>80%); however, the viable areas demonstrated fluorescence with a strong association with areas of TTF-1 and CEACAM5 and a lack of CK 5/6 and p63 staining, indicating adenocarcinoma spectrum lesions. Similarly, CEACAM5 staining was associated with SGM-101 fluorescence emission in this cohort (eFigure 7 in Supplement 2). Four of 5 patients in this cohort had lesions deeper than 12 mm from the pleural surface, indicating a lack of in vivo fluorescence (1.2 cm in patient 7 vs 0.33 cm in patient 8). SGM-101 allowed the assessment of CEACAM5-positive nodules, margins, and normal tissue (eFigures 7–9 in Supplement 2). Malignant nodules with the highest antigenic concentration of CEACAM5, as demonstrated with immunohistochemical staining, had the highest fluorescence emission compared with the margin of the tumor and normal lung and distant normal lung. In addition, none of the lymph nodes examined during the procedures demonstrated fluorescence, which was concordant with final pathology demonstrating the absence of lymphatic metastases.

## Discussion

Intraoperative molecular imaging–guided resections have been increasingly added to the armamentarium of solid organ malignant neoplasm resections. However, malignant tumors, particularly in the lung, are heterogonous, which requires exploration of different cellular targeting agents. One such target we explored in this study was the CEACAM5 cell surface glycoprotein, which is expressed in various adenocarcinomas, including colon, rectum, pancreas, esophagogastric, and lung adenocarcinomas.^16^ We hypothesized that SGM-101, which is an anti-CEACAM5 antibody–conjugated fluorochrome, will specifically fluorescently label CEACAM5-positive lung nodules. To our knowledge, this is the first study in the literature that explores CEACAM5 glycoprotein as a potential target in IMI-guided lung nodule resections.

The feasibility and applicability of IMI tracers rely significantly on the clinical utility and ability to use the product in practice daily. Although the CEACAM5 glycoprotein has been extensively studied and validated in the management of various gastrointestinal malignant neoplasms, the same cannot be said in lung cancer.^21,25^ Literature reports^17,18,23,27,28^ demonstrate variable expression of the glycoprotein in lung cancer, and significant geographic variables are associated with the data. Therefore, we initially wanted to explore CEACAM5 as a potential target in our overall patient population.

Twenty-two of 33 lung adenocarcinomas (67%) analyzed in our cohort demonstrated CEACAM5 expression by immunohistochemistry, and glycoprotein expression was largely detected in the tumor core itself rather than adjacent normal tissue, which is significant in developing targeted tracers to avoid false positivity (eFigures 1 and 2 and eTable 1 in Supplement 2). Analysis of a large national cancer database similarly demonstrated NSCLC to have a high messenger RNA expression of CEACAM5 compared with normal and in line with pancreatic, gastric, and esophageal cancers for which glycoprotein is an established clinical diagnostic and prognostic marker (eFigure 3 in Supplement 2).^16,29^ In addition, although the sample size was small, higher CEACAM5 expression was noted in higher-stage disease.

In the control group of patients with metastatic pulmonary nodules, SGM-101 allowed for the in vivo and ex vivo localization of CEACAM5-positive tissues (eTable 6 in Supplement 2). Serum CEA levels had a good positive predictive value, but negative or normal values did not necessarily exclude fluorescence detection (Figure 1 and Figure 2). These results corroborate previous findings of SGM-101 and indicate the glycoprotein targeting and specificity of the antibody fluorochrome conjugate, which does not change behavior in the pulmonary parenchyma.^30^ The CEACAM5 expression levels at index primary lesion resection also appear to be correlated with metastatic CEACAM5 expression, which can further inform thoracic surgeons about the feasibility of using SGM-101 during metastasectomy.

With the observation that lung parenchyma does not hinder SGM-101’s ability to penetrate pulmonary parenchyma, we explored NIR tracer lesion labeling in patients with pulmonary nodules suggestive of disease. It is our routine institutional practice not to perform diagnostic biopsy for patients with lesions concerning for primary lung cancer and unlike the control cohort did not have preoperative CEACAM5 or diagnosis status. Nevertheless, none of the 2 patients with benign tumors had any fluorescence in vivo, ex vivo, or in histologic assessment. This finding was associated with a lack of CEACAM5, TTF-1, CK 5/6, p63, and Ki-67 staining (Figure 3 and Figure 4).

Conversely, patients with strong CEACAM5 expression demonstrated fluorescence similar to that of the control group. SGM-101 could be detected in ex vivo analysis colocalized with CEACAM5 staining. Tumors with no CEACAM5 expression as predicted did not demonstrate any fluorescence in any setting. Unlike the control group, most lesions were smaller than 0.75 cm and tended to be deeper in the pulmonary parenchyma. The depth of lesion localization is a known challenge for NIR tracers and correlates with the emission wavelength of the target fluorochrome. With the increasing developments of new fluorochromes that operate in the NIR-II range (>1000 nm), it is expected that deeper parenchyma in solid organ malignant neoplasms can be explored. We demonstrated that the targeted antibody in SGM-101 is highly specific and can potentially be conjugated to a different validated fluorochrome once NIR-II tracers become accessible.

### Limitations

There are several limitations that need to be addressed in this proof-of-principle study. The sample size in each arm of the study was small, with a total of 9 patients with 14 total lesions undergoing SGM-101–guided resection. Larger clinical phase 2 and 3 randomized controlled studies are needed to discern the benefit of SGM-101 in IMI-guided lung cancer resections. In addition, in this early exploratory cohort, the selection of patients with positive serum CEA levels and/or preoperative biopsy with CEACAM5 immunohistochemical assessment can identify optimal patients who would benefit from SGM-101. Ideal patients who could benefit would be those with lesions greater than 1 cm that are closer to the pleural surface. Although the tracer adds additional cost to surgical care, potential benefits can be derived from SGM-101–guided resections. In optimally selected patients, the NIR tracer can confirm the detection of primary tumors, assess margins, and potentially identify occult lesions that would otherwise be missed by conventional means. These benefits would also facilitate surgery progression in cases of lesion fluorescence because the surgeon would not have to wait for frozen section confirmation, which can take up to 1 hour in certain facilities, therefore decreasing the time the patient is under anesthesia and operating room costs. Additional trials with larger cohorts can address these claims and challenges.

## Conclusions

This proof-of-principle, phase 1 nonrandomized controlled trial demonstrates that the CEACAM5-targeted NIR tracer SGM-101 can detect CEACAM5 glycoprotein–positive lung tumors. Ideal patients are those with lesions larger than 1.0 cm who are CEACAM5 positive or patients with positive serum CEA levels. Additional, larger-scale trials are needed to validate the study findings, but the current results demonstrate that CEACAM5 surface glycoprotein targeting is a feasible target with clinical utility.

## Supplementary Material

Supp 3 Data Sharing Statement
CEACAM5 Receptor–Targeted Intraoperative Molecular Imaging to Detect Lung Cancer. The Video shows the use of carcinoembryonic antigen–related cell adhesion molecule type 5 (CEACAM5) receptor–targeted intraoperative molecular imaging on (A) a CEACAM5-positive tumor, (B) a benign, CEACAM5-negative nodule, (C) a CEACAM5-positive tumor in situ, and (D) a bisected CEACAM5-positive tumor.

Supp 1 Trial Protocol

Supp 2 **eMethods.** Supplemental Methods
**eTable 1.** Fisher’s Exact Test Exploring CEACAM5 Expression With Respect to Patient/Histopathologic Characteristics in 33 Patients Who Underwent Resection of Lung Adenocarcinoma
**eTable 2.** Demographic and Histopathologic Characteristics of the 10 Patients Included in the Study
**eTable 3.** List of Complications Observed in the Patient Cohort for the Study
**eTable 4.** Histopathologic Details of Patients in the Control Group With Corresponding SGM-101-Related Intraoperative Findings
**eTable 5.** Primary Malignancy Surgical Characteristics of the Patients in the Metastasis Control Group
**eTable 6.** Characteristics of SGM-101 Guided Resections of the Lung Nodules in the Cohort
**eFigure 1.** CEACAM5 Core Biopsy IHC Staining of 33 Consecutive Lung Adenocarcinoma Patients With (1) Cancer Tissue, (2) Cancer Adjacent Normal, and (3) Distant Normal Lung Demonstrating 22/33 Positive CEACAM5 Presence
**eFigure 2.** Higher-Stage Lung Adenocarcinoma Correlated With an Increased Rate of CEACAM5 Expression (AJCC 8^th^ ed)
**eFigure 3.** TCGA Database Analysis Demonstrates CEACAM5 Expression in Various Prevalent Solid Organ Malignancies, With Colorectal Adenocarcinoma Demonstrating the Highest mRNA Expression (16 Log2(Value +1)) and Melanoma Demonstrating the Lowest Median Expression (1 (Log2(Value +1))
**eFigure 4.** Patients 3 and 4 With Normal Serum CEA Levels With SGM-101 Ex Vivo Localization of Subcentimeter Nodules
**eFigure 5.** SGM-101 Fluorescence Correlates With CEACAM5 Presence by IHC (Patient 3)
**eFigure 6.** SGM-101 Localization to CEACAM5+ Areas
**eFigure 7.** CEACAM5 IHC and Serum CEACAM5/CEA Can Predict SGM-101 Fluorescence Success and Tumor Localization
**eFigure 8.** Intraoperative Findings for Patient 2 in the Metastatic Control Cohort During SGM-101 Guided Lung Nodule Resection
**eFigure 9.** Overview of CEACAM5-Targeted Fluorescence Localization of Lung Nodules With SGM-101

## Figures and Tables

**Video:** CEACAM5 Receptor–Targeted Intraoperative Molecular Imaging to Detect Lung Cancer. The video shows the use of carcinoembryonic antigen–related cell adhesion molecule type 5 (CEACAM5) receptor–targeted intraoperative molecular imaging on (A) a CEACAM5-positive tumor, (B) a benign, CEACAM5-negative nodule, (C) a CEACAM5-positive tumor in situ, and (D) a bisected CEACAM5-positive tumor.

## Key Points

### Question

Can SGM-101, a carcinoembryonic antigen–related cell adhesion molecule type 5 (CEACAM5) receptor–targeted intraoperative molecular imaging fluorophore, be used for CEACAM5-positive metastatic and primary lung tumors?

### Findings

In this nonrandomized controlled trial with 10 patients, SGM-101 localized in situ, ex vivo, and on fluorescence microscopy to CEACAM5 glycoprotein expressing gastrointestinal lung metastases as well as primary lung adenocarcinomas.

### Meaning

Although CEACAM5 has been extensively studied in gastrointestinal malignant neoplasms, these findings suggest that the glycoprotein can be a suitable target for molecular imaging–guided localization in thoracic oncology.
