# Supplementary material for: Carcinoembryonic Antigen-Related Cell Adhesion Molecule Type 5 Receptor-Targeted Fluorescent Intraoperative Molecular Imaging Tracer for Lung Cancer: A Nonrandomized Controlled Trial
Source: JAMA Netw Open. Author manuscript; Available in PMC 2024 Jan 3. (PMC10292762; doi:10.1001/jamanetworkopen.2022.52885)
Supplement: Supp 3 — Data Sharing Statement CEACAM5 Receptor–Targeted Intraoperative Molecular Imaging to Detect Lung Cancer. The Video shows the use of carcinoembryonic antigen–related cell adhesion molecule type 5 (CEACAM5) receptor–targeted intraoperative molecular imaging on (A) a CEACAM5-positive tumor, (B) a benign, CEACAM5-negative nodule, (C) a CEACAM5-positive tumor in situ, and (D) a bisected CEACAM5-positive tumor. [file NIHMS1901881-supplement-Supp_3.pdf]

Data Sharing Statement

Azari. Carcinoembryonic Antigen-Related Cell Adhesion Molecule Type 5 Receptor-Targeted Fluorescent Intraoperative Molecular Imaging Tracer for Lung Cancer. *JAMA Netw Open*. Published January 27, 2023. doi:10.1001/jamanetworkopen.2022.52885

Data
Data available: No

Additional Information
Explanation for why data not available: All data in this phase I study is available in the manuscript and supplementary data. Data is available upon request from corresponding author.
