# Supplementary material for: Carcinoembryonic Antigen-Related Cell Adhesion Molecule Type 5 Receptor-Targeted Fluorescent Intraoperative Molecular Imaging Tracer for Lung Cancer: A Nonrandomized Controlled Trial
Source: JAMA Netw Open. Author manuscript; Available in PMC 2024 Jan 3. (PMC10292762; doi:10.1001/jamanetworkopen.2022.52885)
Supplement: Supp 1 — Trial Protocol [file NIHMS1901881-supplement-Supp_1.pdf]

V

CLINICAL RESEARCH PROTOCOL

INTRAOPERATIVE MOLECULAR IMAGING OF PULMONARY NODULES BY SGM-101, A FLUOROCHROME-LABELED ANTI-CARCINO-EMBRYONIC ANTIGEN (CEA) MONOCLONAL ANTIBODY

**Regulatory Sponsor:**	Sunil Singhal, M.D.<br>Department of Surgery<br>Perelman School of Medicine<br>3400 Spruce Street<br>6 White Building<br>Philadelphia PA, 19104
**Principal Investigator:**	Sunil Singhal, MD
**Funding Sponsor:**	Center for Precision Surgery<br>Abramson Cancer Center
**Study Product:**	SGM-101 is a CEA-specific chimeric antibody conjugated with a NIR emitting fluorochrome
**Protocol Number:**	UPCC 02519
**IND Number:**	145137

Version:

1.0	1/14/19
1.1	11/1/19
1.2	12/23/19
2.0	05/05/2020

TABLE OF CONTENTS

TABLE OF CONTENTS 2

LIST OF ABBREVIATIONS 4

STUDY SUMMARY 5

SCHEDULE OF PROCEDURES 6

1 INTRODUCTION 7

- 1.1 BACKGROUND 7
- 1.2 INVESTIGATIONAL AGENT 8
- 1.3 PRECLINICAL DATA 10
- 1.4 CLINICAL DATA 11

2 STUDY OBJECTIVES 14

3 STUDY DESIGN 15

- 3.1 GENERAL DESIGN 15
- 3.2 STUDY ENDPOINTS 17
  - *Efficacy* *17*
  - *Safety* *17*

4 SUBJECT SELECTION AND WITHDRAWAL 17

- 4.1 INCLUSION CRITERIA 17
- 4.2 EXCLUSION CRITERIA 17
- 4.3 SUBJECT RECRUITMENT AND SCREENING 18
- 4.4 EARLY WITHDRAWAL OF SUBJECTS 18
  - *When and How to Withdraw Subjects* *18*
  - *Data Collection and Follow-up for Withdrawn Subjects* *19*

5 STUDY DRUG 19

- 5.1 DESCRIPTION 19
- 5.2 TREATMENT REGIMEN 19
- 5.3 METHOD FOR ASSIGNING SUBJECTS TO TREATMENT GROUPS 19
- 5.4 PREPARATION AND ADMINISTRATION OF STUDY DRUG 19
- 5.5 SUBJECT COMPLIANCE MONITORING 19
- 5.6 PRIOR AND CONCOMITANT THERAPY 20
- 5.7 PACKAGING 20
- 5.8 BLINDING OF STUDY DRUG 20
- 5.9 RECEIVING, STORAGE, DISPENSING AND RETURN 20

6 STUDY PROCEDURES 20

- 6.1 VISIT 1- INITIAL CONSULT (UP TO 90 DAYS BEFORE SURGERY) 20
- 6.2 VISIT 2- CHPS(INFUSION UP TO 5 DAYS BEFORE SURGERY) 21
- 6.3 VISIT 3- SURGERY 21

7 STATISTICAL PLAN 22

- 7.1 SAMPLE SIZE DETERMINATION 22
- 7.2 STATISTICAL METHODS 23

8 SAFETY AND ADVERSE EVENTS 23

- 8.1 DEFINITIONS 23

Unanticipated Problems Involving Risk to Subjects or Others *23*
Adverse Event *24*
Serious Adverse Event *24*
Adverse Event Reporting Period *24*
Preexisting Condition *25*
General Physical Examination Findings *25*
Post-study Adverse Event *25*
Abnormal Laboratory Values *25*
Hospitalization, Prolonged Hospitalization or Surgery *26*
8.2 RECORDING OF ADVERSE EVENTS 26
8.3 REPORTING OF SERIOUS ADVERSE EVENTS AND UNANTICIPATED PROBLEMS 26
Investigator reporting: notifying the Penn IRB *27*
Investigator reporting: notifying the Pharmacovigilance company *27*
Reporting Process *27*
Reporting Deaths: more rapid reporting requirements *28*
Other Reportable events: *28*
8.4 REPORTING SAE'S TO THE DSMC 29
8.5 UNBLINDING PROCEDURES 29
8.6 STOPPING RULES 29
8.7 MEDICAL MONITORING 29

9 DATA HANDLING AND RECORD KEEPING 30

9.1 CONFIDENTIALITY 30
9.2 SOURCE DOCUMENTS 30
9.3 CASE REPORT FORMS 30
9.4 RECORDS RETENTION 31

10 STUDY MONITORING, AUDITING, AND INSPECTING 31

10.1 STUDY MONITORING PLAN 31
10.2 AUDITING AND INSPECTING 31
10.3 REPORTING OF EXCEPTIONS AND DEVIATIONS 31
Exception *31*
Deviation *32*

11 ETHICAL CONSIDERATIONS 32

12 STUDY FINANCES 33

12.1 FUNDING SOURCE 33
12.2 CONFLICT OF INTEREST 33

13 PUBLICATION PLAN 33

14 ATTACHMENTS 33

15 REFERENCES 33

List of Abbreviations

PI- Principal Investigator
SAE- Serious Adverse Event
API- Active Pharmaceutical Ingredient
CHPS- Center for Human Phonemic Science (Formerly CTRC- Clinical Translational Research Center)

Study Summary

Title	INTRAOPERATIVE MOLECULAR IMAGING OF PULMONARY NODULES BY SGM-101, A FLUOROCHROME-LABELED ANTI-CARCINO-EMBRYONIC ANTIGEN (CEA) MONOCLONAL ANTIBODY
Short Title	Intraoperative Imaging of Pulmonary Nodules by SGM-101
Protocol Number	xxxxx
Phase	Pilot and exploratory
Methodology	Open Label
Study Duration	24 Months
Study Center(s)	Single Center
Primary Objective	The primary end-point of the study is to determine the sensitivity of SGM-101 in identifying lung nodules when excited by an near-infrared light source.
Secondary Objective	The secondary objective is to measure the concordance of fluorescence and tumor type.
Number of Subjects	We intend to enroll 20 lung cancer subjects in this study.
Diagnosis and Main Inclusion Criteria	The study is focusing on subjects presenting with suspected malignancies of the lung and pleura who are considered to be good surgical candidates.
Study Product, Dose, Route, Regimen	SGM-101 is a fluorescent conjugate comprised of a tumor-specific, anti-carcinoembryonic antigen (CEA) monoclonal antibody and a near infrared (NIR) emitting fluorochrome. We will give 5 to 10 mg intravenously 3 to 5 days prior to surgery.
Duration of administration	The proposed dose of SGM-101 to be investigated in this study is 10 mg to be administered intravenously over 30-60 minutes.
Reference	Non-placebo controlled
Statistical Methodology	The sensitivity and specificity of the intra-operative imaging will be estimated using microscopic pathological examination as the gold standard. Exact 95% confidence intervals with be generated around these estimates.

V

Schedule of Procedures

Study Procedure	Visit 1 Screening	Visit 2 Infusion Day	Visit 3 Surgery Day	Visit 4 Post-Operative F/up
Informed consent	X			
Inclusion/exclusion criteria met	X			
Lung, pleural nodule or mass presumed to be resectable on pre-operative assessment	X			
Medical history	X			
Serum or urine pregnancy test for childbearing women		X		
Vital signs (Temp, HR, BP, Pulse/Ox, Skin Color)		X		
Study drug administration		X		
Surgery with associated procedures including intra-operative imaging			X	
AE assessments		X	X	X
ADE assessments		X	X	X

1 Introduction

This document is a protocol for a human research study. This study is to be conducted according to US and international standards of Good Clinical Practice (FDA Title 21 part 312 and International Conference on Harmonization guidelines), applicable government regulations and Institutional research policies and procedures.

1.1 Background

According to the World Health Organization, lung cancer is the most common cause of cancer-related death in men and women, and is responsible for 1.5 million deaths worldwide annually as of 2017. Surgery remains the best option for subjects presenting with operable Stage I or II cancers, however the five-year survival rate for these candidates remains at a dismal 73% for Stage I and 53% for Stage II (1). The high rates of local recurrence suggest that surgeons are unable to completely detect and remove primary tumor nodules in a satisfactory manner as well as lingering metastases in sentinel lymph nodes. By ensuring a negative margin through imaging during surgery it would be possible for us to improve the rates of recurrence free subjects and thus overall survival.

Over the last several years, our group has taken a novel approach to the surgical approach (2-5). Specifically, we have started injecting contrast agents that go selectively to tumors and allow them to fluoresce. We postulate that provides an additional tool in the operating room for surgeons to find tumors and their margins.

Thoracic malignancies are the ideal disease to investigate intra-operative imaging. Over 25-65% of lung and pleural malignancies express carcinoembryonic antigen (CEA), therefore making CEA a target for imaging agents (6-11). Interestingly, there is minimal expression of CEA in normal lung, therefore, the background to noise of CEA in cancers versus normal lung is very high. Unfortunately, unlike colorectal cancer, it is not evident that serum CEA levels correlate with lung cancer, specifically non-small cell lung cancer.

CEA is a glycoprotein product of the gene CEACAM-5 and is a member of the immunoglobulin superfamily that serves as a cell-adhesion molecule and may also have an effect on innate immunity. High serum CEA levels have been identified as a prognostic factor in both resected NSCLC and in metastatic disease (6, 7, 11-13).

CEA has previously been considered a promising target for monoclonal antibodies to be used in radio-immunodiagnostic and radio-immunotherapy, and over a hundred clinical studies involving a number of different anti-CEA antibodies have been conducted in this field (14, 15). The radiolabeled anti-CEA antibody CEA-Scan (arcitumomab) from Immunomedics GmbH was authorized in the European Union in 1996 and in the United States in 1999 to assess recurrent or metastatic disease in patients with colorectal cancer. The product has since been withdrawn from the market for commercial reasons but it is important to mention that during its 9 years of use, no adverse effects have been reported.

V

1.2 Investigational Agent

(see Investigator’s Brochure)

Class

In vivo diagnostic agent.

Structure

The SGM-101 active ingredient is a covalent conjugate of the SGM-Ch511 anti-CEA chimeric monoclonal antibody with the fluorochrome BM-104 (Figure III.1-1). The BM-104 fluorochrome is conjugated to free amino groups of the antibody via an amide bond.

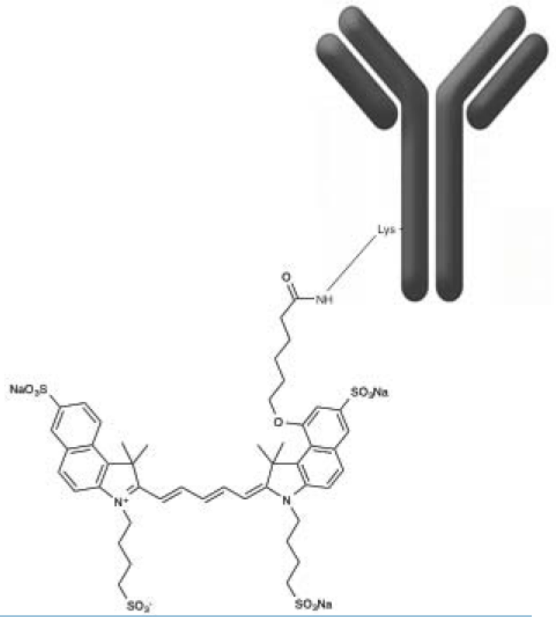

Figure Schematic representation of the SGM-Ch511-BM-104 conjugate.

The SGM-101 drug substance presents as a mixture of fluorochrome-antibody conjugate species of different conjugation levels, ranging from 1 to 6 BM104 molecules per antibody and of unconjugated species. The human constant domains of the antibody are of the G1m3 allotype for the heavy chains and Km3 for the light chains.

General properties

The SGM-Ch511-BM-104 conjugates are characterized by peaks of absorbance at 280 nm (antibody) and 685 nm (fluorochrome).
Excitation and emission wavelengths peaks of SGM-101 are 686 and 704 nm, respectively.

Physical form

The SGM-101 drug substance presents as a 5.0 mg/mL solution of the active ingredient in 10mM KH2PO4, 10 mM Na3citrate, 300 mM arginine, 0.02% Tween-20, pH 6.0.

Non-Clinical Pharmacology, Pharmacokinetics And Toxicology

Pharmacology

Non-clinical pharmacology studies have demonstrated SGM-101's efficacy in binding to human CEA expressing cells *in vivo* in terms of specificity, sensitivity and targeting scope using four different xenografted or transgenic mouse models.

SGM-101 allowed *in vivo* identification of tumor nodules overexpressing CEA in all models tested, regardless of the location of tumors (including in the peritoneal cavity in peritoneal carcinomatosis mouse models, subcutaneously, in the cecum, pancreas or breast in orthotopic tumor models and in the liver in metastatic models) and of their origin (colorectal*,* pancreas or breast), confirming the targeting ability of the anti-CEA antibody.

In addition, a high level signal-to-noise ratio allows the detection of very small nodules invisible to the naked eye, even for an experienced investigator.

Moreover, GLP-compliant safety pharmacology studies demonstrated that intravenous SGM-101 administration did not lead to any adverse effects neither on the central and peripheral nervous systems in Wistar rats, nor on the cardiovascular and respiratory functions in Beagle dogs at dose levels of up to 40 mg/kg (240 mg/m2) and 20 mg/kg (400 mg/m2), respectively.

Pharmacokinetics and metabolism

In vitro stability studies in human plasma at 37°C have confirmed the stability of the amide bonds linking the BM-104 fluorochrome to the antibody.

In vivo studies conducted in human CEA-expressing mouse models have shown similar pharmacokinetic profiles as in wild type strains; CEA expression in this transgenic model is exclusively apical, with the antigen not being accessible to circulating SGM-101. Tissue distribution was also comparable in transgenic human CEA-expressing mice and in wild type animals.

SGM-101 was shown to be rapidly eliminated in immunosuppressed mice bearing human intraperitoneal tumors overexpressing CEA and in normal Swiss mice, with remaining SGM-101 levels of less than 10 % of the injected dose, 10 days after injection.

Toxicokinetic evaluation performed in Wistar rats treated intravenously at 5, 20 or 40 mg/kg/day showed that plasma SGM-101 levels increased with dose in an almost

proportional manner. Exposure of SGM-101 was slightly higher in males than in females at the highest dose level.

Preliminary analysis of the pharmacokinetic data from the FIH clinical trial indicates that SGM-101 maximal serum concentrations are usually reached at or close to the end of the infusion and decrease progressively thereafter, with a mean t1/2 of approximately 30 h regardless of the dose level (range of values between 16.9 and 36.3 h). From 5 to 12.5 mg, exposure tends to increase with dose but appears to remain unchanged between 12.5 and 15 mg.

Safety

Toxicity studies conducted with SGM-101 in Wistar rats and in Beagle dogs showed that the drug is well tolerated up to the maximal dose tested of 40 mg/kg per day (85-fold the intended maximum clinical dose) in rats and 5 mg/kg (10-fold the intended maximum clinical dose) in dogs, with minor and transient signs of an infusion reaction at higher dose levels in the latter.

A tissue cross-reactivity study of SGM-101 conducted on a panel of 42 human frozen tissues and blood smears confirmed on-target only binding of SGM-101. BM-104 fluorochrome did not exhibit any signs of toxicity at 3.4 mg/kg (140-fold the maximal anticipated clinical exposure) in Wistar rats and was non-mutagenic in the Ames test.

To date no adverse event related to the study drug has been reported in the ongoing clinical trials, at dose levels ranging from 5 to 15 mg.

1.3 Preclinical Data

(See Investigator's Brochure)

The first use of a fluorescent anti-CEA antibody conjugate was described by the team of Andre Pelegrin under *ex vivo* conditions in patients with colorectal tumors, opening the possibility of such immunodetection in the clinical field (16). Further studies allowed the detection of submillimeter tumor nodules in a murine peritoneal carcinomatosis model (17).

Preclinical evaluation of several CEA-targeting near-infrared fluorescent tracers has been described in the past few years, displaying a good tumor-binding of the tracers (18, 19). Complete resection of human colon cancer was also achieved by fluorescence-guided surgery in an orthotopic nude mouse model, resulting in decreased recurrence and increased survival (19). Fluorescence-guided surgery using a fluorophore-conjugated-anti-CEA antibody to visualize tumors also showed promising results in preclinical orthotopic models of human pancreatic cancer, with

both an improvement of surgical resection and an increased survival of the animals (20).

SGM-101 is a CEA-specific chimeric antibody conjugated to a fluorophore emitting NIR fluorescence around 700 nm. After injection to the patient prior to surgery, the antibody conjugate binds to CEA-overexpressing tumor tissue. By using a NIR fluorescence imaging system, the surgeon can visualize the tumor and detect even small-size nodules invisible to the naked eye, enabling real-time guidance during surgery.

Preclinical studies showed that SGM-101 binds to CEA-positive CRC-cells following intravenous administration and that even small peritoneal metastases can be visualized with a dedicated NIR imaging system, suggesting it to be a worthwhile candidate for clinical application (21).

SGM-101 offers major diagnostic improvements compared to routine surgery such as the clear delineation of tumor masses within a normal environment, the detection of a subclinical carcinomatosis in high-risk patients, or the assessment of residual disease allowing an adapted postoperative treatment.

These properties could enable a more complete surgical resection of tumor lesions and subsequently greatly enhance the patient’s prognosis.

1.4 Clinical Data

To date the efficacy of SGM-101 has been evaluated in 2 clinical studies: a first-in-human trial that was initiated in France (SGM-CLIN01) in advanced cancer patients presenting with peritoneal carcinomatosis originating from gastrointestinal malignancies, and a second trial (SGM-CLIN02), in the Netherlands, for the delineation of pancreatic and colorectal tumors. Patient enrollment is complete for the SGM-CLIN02 study. These clinical studies are further described below.

Clinical Phase I: Safety Of SGM-101 In Patients With Peritoneal Carcinomatosis From CEA Overexpressing Gastrointestinal Cancer (Study # SGM-CLIN01 / Protocol # ICM-URC-2014/35 / # EudraCT : 2014-004017-87.)

A first-in-man open-label Phase I study was conducted in Montpellier, France (protocol N°ICM-URC-2014/35) to assess the safety of a single intravenous administration of SGM-101 in patients with peritoneal carcinomatosis from CEA-over-expressing gastrointestinal cancers.

Patients were enrolled according to a 3+3 dose escalation scheme at five dose levels: 5 mg, 7.5 mg, 10 mg, 12.5 mg and 15 mg (cohorts 1 to 5). Patients were administered SGM-101 24 hours prior to surgery, and an additional treatment group at the 15 mg dose level was included in order to assess administration 48 hours prior to surgery (cohort 5 pts).

Fifteen patients were enrolled in the study as part of the dose escalation (3 per dose level). Three additional patients were enrolled at the 15 mg dose level according to the second administration scheme (*i.e*. 48 hours prior to surgery).

Visibility and background were assessed by two different and independent surgeons using fluorescent SGM-101. The two surgeons evaluated the peritoneal carcinomatosis index (PCI) without then with fluorescence, to check for the presence of additional fluorescent lesions. However, only very few efficacy data were reported in the study. Only 8 out of 18 patients underwent resection of carcinomatosis because some patients were considered unresectable during surgery due to the extent of carcinomatosis and other patients were not resected because no peritoneal carcinomatosis was confirmed during surgery, whether without or with fluorescence. And, of the 8 patients who were resected, only 3 were evaluable for efficacy due to problems with the NIR camera used in this first study so that given this small sample no conclusion was reached in regard to efficacy.

Clinical Phase I/II : Safety of SGM-101 in Patients with Cancer of the Rectum or Pancreas (Study # SGM-CLIN02 / Protocol # CHDR1517 / EudraCT # : 2015-003281-88)

This open-label, ascending single dose study was performed to explore the safety, tolerability, pharmacokinetics and performance of SGM-101 in patients with colorectal and pancreas cancer. This study was a collaborative project between the department of surgery of Leiden University Medical Center (LUMC), Catharina Hospital Eindhoven (CZE), Erasmus University Medical Center (EMC) and the Centre for Human Drug Research (CHDR), The Netherlands. In this report 75 patients (46 males, 29 females), with primary, recurrent or metastasized colorectal cancer (n=61) and pancreas cancer (n=14) were included and analyzed. Five different doses of SGM-101 were evaluated: 5 mg (7 patients), 7.5 mg (10 patients), 10 mg (39 patients), 12.5 mg (9 patients) and 15 mg (10 patients) and dosed at least 24 hours before the planned surgery. The choice for period of at least 24 hours between dosing and surgery was based upon the observations in animals that showed that sufficiently strong signals were reached between 24 and 72 hours. In the study the dosing period varied between 2-6 days before the scheduled surgery, where 4 days prior to surgery showed the most favorable results.

Overall, all doses up to 15 mg SGM-101 were well-tolerated. None of the included patients experienced an allergic reaction or TEAE that was considered of clinical importance or led to discontinuation of the study. No trends or changes of clinical importance were noted in the vital signs, ECG, or laboratory parameters after SGM-101 administration. Hence, there appears to be no safety concerns associated with iv administration (30 minutes infusion) of SGM-101 at doses up to 12.5 mg in pancreas patients and 15 mg in colorectal cancer patients.

The performance of SGM-101 in the study was evaluated on the basis of the obtained fluorescence imaging results during surgery and the concordance with the pathology.

In the study, a clear benefit of using SGM-101 was found in the colorectal cancer patients where SGM-101 was beneficial in the identification of additional lesions that were clinically not suspect for malignancy. In the colorectal patients, additional lesions were found in 8 patients. A total of 21 additional lesions were identified by using intraoperative fluorescence; 20 lesions in the 10 mg SGM-101 group (dosed 4 days prior to surgery) and 1 lesion in the 12.5 mg SGM-101 group (dosed 4 days prior to surgery). The mean TBR of these additionally identified lesions was 1.7. Importantly, these lesions were confirmed as malignant during pathology review.

The analyzed images showed that the mean intraoperative TBR had the highest mean value of 1.7 in the 10 mg SGM-101 dose group when compared to the other dose levels. Higher dose did not always result in a higher TBR, most likely because the background fluorescence signal was also increased. Furthermore, the results show that the dose level 10 mg SGM-101 has the most optimal sensitivity (97%) and specificity (52%) combination along with an adequate negative predictive value (93%). A possible shortcoming of the study is that the amount of patients included in each dose level is not equal resulting in a sub-optimally powered comparison. The additional cohort which was implemented to evaluate a delay in the injection time to 6 days prior to surgery did not result in higher TBR values.

A noteworthy finding is that 10 mg SGM-101 has a high negative predictive value (93%). This opens the possibility that clinical decisions to resect tissue can be changed based on the (fluorescent) findings during surgery. Nevertheless, during these clinical decisions the possibility for false negatives should always be taken into account, even though the study only had 3 false negatives; 3 of the 184 lesions (~ 2%).

In the pancreas cancer patients, intraoperative fluorescence imaging of pancreas cancer was feasible as fluorescence could be detected in the primary tumors as well as the metastases. Despite the suboptimal intrinsic characteristics of pancreas cancer, including poor vascularization, the results showed that SGM-101 can reach and bind to CEA-expressing tumor cells. The TBRs in the pancreas cancer patients were more ambiguous when compared to the CRC patients. This can possibly be explained by the histopathology of pancreas cancer, with solitary ducts of tumor cells in preexistent normal pancreatic tissue and a remarkable desmoplastic stroma, which could make the fluorescence pattern more sparse than with other tumor types. Most of the pancreas cancer patients were dosed with 7.5 mg SGM-101 and the highest mean intraoperative TBR was calculated in the 5mg SGM-101 group.

The calculated TBRs provided insight on the performance of the different dose levels studied in CRC and pancreas cancer patients. It is considered that the intraoperative TBR should be leading, as the decision making to resect tissue (based on

fluorescent status or not) is done during surgery. However, decision making on intraoperative fluorescence only has its drawbacks for instance for deep-seated tumors that cannot readily be visualized because of the limited penetration depth of fluorescent light (maximally 1 cm) or for tumors that are in an anatomic position that precludes proper imaging. Additionally, overlying tissue and blood in the surgical field can also cause intraoperative false-negativity. It is therefore that ex-vivo (back table) results should also be taken into account. In the majority of lesions, fluorescence was not visible or measured intraoperatively, but fluorescence could be clearly detected after resection, which was subsequently confirmed to be malignant by pathology review. In these situations, fresh frozen sections are of added value.

It was therefore decided to present the final conclusion on concordance between pathology results and the combined fluorescence results (in-vivo and ex-vivo fluorescence). Several considerations should be taken into account when using this approach. Combining in vivo and ex vivo imaging to assess concordance may diminish the chance that a finding is labelled false negative, while it is not detected during the surgical procedure itself. However, as back table (ex vivo) results may provide important information during the surgery on the completeness of resection (margin-assessment), it felt justifiable to follow this approach. In addition, caution should be used when combining in-vivo and ex-vivo results. During back table imaging, surrounding tissue plays a lesser role since only the excised lesion is being assessed, which can possibly influence the fluorescence emitted.

A relative high amount of false positive lesions were found in the CRC patients. Although a definitive explanation is not readily available, it was noted that a majority of these false positives were found in the reproductive organs (such as ovaries and vesicula seminalis). Apparently, these organs show a high binding (absorption) capacity for SGM-101. Why these organs were fluorescent during surgery, without containing tumor cells is not yet explained. There are, however, no indications that gender-based dosing is necessary.

Overall, according to the current descriptive results, the calculated TBRs and concordance show that 10 mg SGM-101 4 days prior to surgery is a reasonable choice and possibly the most favorable treatment for intraoperative fluorescence imaging of patients with colorectal cancer. For pancreas cancer, the highest intraoperative TBR was seen in the 5 mg SGM-101 dose level group. CEA was chosen as a suitable target for fluorescence imaging of colorectal and pancreas cancer. The study confirmed its potential and demonstrated that both colorectal and pancreas cancer, including metastases of both tumor types, can be visualized using intraoperative fluorescence imaging with SGM-101.

2 Study Objectives

Primary Objective

The primary study objectives are to assess the sensitivity and specificity of SGM-101 in detecting non-small cell lung carcinomas during surgery.

3 Study Design

3.1 General Design

Subjects will be seen in a General Thoracic Surgery clinic. If they have a lung nodule that is suspicious for a lung non-small cell lung cancer, they will be a candidate for the operation. From our prior experience, we have an 90% positive predictive value based on CAT and PET scans that a subject with a suspected lung cancer will in fact have lung cancer. The subjects with lung and pleural nodules will be prospectively consented to participate in this trial. There will be no randomization or control group and only subjects previously scheduled to undergo surgery will be eligible to participate. We anticipate a 24-month period will be necessary to reach our accrual goal of 20 subjects.

After obtaining informed consent, subjects will receive a one-time dose of 5 to 10 mg of SGM-101, up to 5 days prior to the planned operation. As a prophylactic measure, we may recommend giving 25 mg of IV Benadryl to the subject prior to the infusion of SGM-101 to ensure possibility of allergic reaction is absolutely minimized. This will be at the discretion of the principal investigator. The goal of surgery in subjects is to remove the nodule and lymph nodes in concern. During surgery, we will take images with an intra-operative camera system. Imaging will take place prior to surgical resection to record the localization of tumors, and post-resection to document the visualization of any residual tumor. All documenting of nodules will take place in an electronic report form.

Our analysis will be the following:

Data Analysis of a Diagnostic Test		Test result		Data Analysis
		True (accept H_0)	False (accept H_A)	
Disease present ("The Truth")	True (H_0)	TP (1 – β)	FN (type 2 error = β = 1-sens)	$sensitivity = \frac{TP}{TP + FN}$
	False (H_A)	FP (type 1 error = α = 1-spec)	TN (1 – α)	$specificity = \frac{TN}{TN + FP}$
Data Analysis		$PPV = \frac{TP}{TP + FP}$	$NPV = \frac{TN}{TN + FN}$	$Accuracy = efficiency = \frac{TP + TN}{TP + TN + FP + FN}$

True positive = fluorescent nodule, pathology is cancer
False positive = fluorescent nodule, pathology is no cancer
True negative = not fluorescent nodule, pathology is no cancer
False negative = not fluorescent nodule, pathology is cancer

Cohort 1: Our first 10 subjects will be a feasibility trial. We will discover whether lung non-small cell lung cancers fluoresce based on the intraoperative images. The surgeon will look at the images during surgery to determine if the tumor is glowing or not. Based on prior experience, we will be cautious for false positives. In our first 10

subjects, if we have 5 or more subjects with a false positive, then we will review the data prior to proceeding. If we have a high false positive rate, then we will likely not proceed because the clinical value of the fluorescent probe is minimal. (Of note, if the first five consecutive patients have false positives, we will stop the study and analyze the data carefully before we will consider proceeding).

Cohort 2: If the first 10 subjects show no significant false positives, then we will perform another 10 subjects. The primary goal will be to determine (a) how many non-small cell lung cancers are detected by intraoperative imaging? Additional data will be collected to determine (b) how deep the imaging system can see into the lung organ, (c) can metastatic cancer cells in lymph nodes and satellite nodules be detected? (d) if the surgeon mistakenly cuts through the tumor, will the intraoperative imaging detect a positive margin? In this cohort, we will not make any clinical decisions to jeopardize the patient or remove significantly more than the intended operation. We will collect more data in this cohort for analysis.

During Imaging, if the removal of a fluorescent lesion does not change the magnitude of the operation, then it can be removed. Until we have more data on the false positive rate of our technology, we will not change the magnitude of the operation based on results from an investigational drug. Lesions that are identified by the surgeon and not the imaging system will be removed at the surgeons' discretion following the standard of care operation.

The duration of surgical procedures to resect thoracic malignancies varies substantially, anywhere from 2- 6 hours or more. It is estimated that visualization of the chest and removal of nodules for the purposes of this study will require an additional ten (10) minutes. Due to potential quenching of the fluorophore, visualization time will be limited to 30 minutes.

The visualization will be conducted with a camera: The Artemis Handheld Camera System-- open-field and laparoscopic-- manufactured by the Quest Medical Imaging. The Artemis has received approval from the FDA for a Phase II ovarian cancer clinical trial in Ohio, The Artemis is registered with the FDA for fluorescence imaging. The Artemis systems are proposed to be non-significant risk (NSR) devices as the devices do not meet the criteria of a significant risk device defined in 21CFR 812.3 (m). Another camera that will be used for visualization is called the Spectropath FLI-10A which is manufactured by Nanjing NuoYuan Medical. The cameras will be stored in the offices in operating room that stores the camera towers and will be used for the surgeries. The camera will be rolled into the operating room by the surgeon performing the case.

There is a chance that being under anesthesia for the additional five (5) to ten (10) minutes could put the subject at an increased risk of having of a common side effect associated with anesthesia. These common side effects include, but are not limited to: nausea and vomiting after surgery, sore throat and hoarseness, shivering/chills, confusion, and muscle aches.

3.2 Study Endpoints

Efficacy

Efficacy will be assessed through determination of the sensitivity and specificity of SGM-101 uptake and expression in identifying non-small cell lung cancer. Additional exploratory efficacy outcomes will include:

- Number of lung nodules lesions identified under usual visual/tactile conditions compared with such lesions identified using SGM-101 and fluorescence imaging
- Number of lymph nodes with metastatic cancer that are identified using visual/tactile conditions compared with such lesions identified using SGM-101 and fluorescence imaging
- Proportion of subjects in whom more lesions are identified using SGM-101 with fluorescence imaging and subsequently confirmed by pathology/immunohistochemistry analysis as cancer compared with using usual visual/tactile conditions to identify such lesions
- Number of cases that surgeon cuts through tumor and the positive margin is detected by the imaging of SGM-101.

Safety

The safety of the study will be determined via incidence rates of all AEs and treatment-emergent AEs (TEAEs) from the time of SGM-101 administration through follow-up.

4 Subject Selection and Withdrawal

4.1 Inclusion Criteria

1. Adult subjects over 18 years of age
2. Subjects presenting with a lung, pleural nodule or mass presumed to be resectable on pre-operative assessment
3. Good operative candidate
4. Subject is capable of giving informed consent and participating in the process of consent.

4.2 Exclusion Criteria

1. At-risk subject populations
    a. Homeless subjects
    b. Subjects with drug or alcohol dependence

c. Children and neonates
d. Subjects unable to participate in the consent process.

2. Female patients should not be pregnant or lactating.. Women of child-bearing potential will be included provided that they have a negative pregnancy test or provide documentation of sterilization, menopausal or post-menopasusal status, prior to infusion.
3. Patients who have received SGM-101 in the past.
4. Patients who have received any investigational drug four weeks of the injection.

4.3 Subject Recruitment and Screening

Subject recruitment will be performed by the surgeon with subjects under his or her direct care. All eligible candidates will be invited to participate in the research in the following manner:

1) During the subject's initial surgical consultation, the surgeon or a member of the study team will describe the study and its risks and benefits. P.I., surgeon on the protocol, or a member of the study team will obtain consent from subject.

2) The day of the subject procedure, 25-50 mg of IV Benadryl may be recommended prophylactically but not required, and the study drug will be administered in the Center for Human Phenomic Research by the PI or CHPSpersonnel. The decision for Benadryl will be at the discretion of the principal investigator.

4.4 Early Withdrawal of Subjects

When and How to Withdraw Subjects

Subjects will be withdrawn from the study if one of the following considerations has been met:

1. The subject decides that they do not wish to have their surgery
2. The subject experiences a SAE or an AE during the injection which would require an intervention of steroids or epinephrine in the OR.
3. The subject withdraws consent.
4. The PI, or a member of the study team, decides that it is not in the subjects' best interest to continue.
5. The subject is non-compliant with the protocol.
6. Lost to follow up due to death

If the subject withdraws, at any time, the reason(s) will be recorded on the relevant page of the case report form. If the subject is discontinued due to AEs or ADEs, the

subject will be monitored until resolution or stability of the event based on the judgment of the investigator.

Data Collection and Follow-up for Withdrawn Subjects

If the subject decides to not participate in the study before their injection, it would make no impact on our study outcomes for survival data, as there is only a single procedure point. However, if the subject wishes to withdraw consent after the study, efforts will be made to secure permission to follow-up with the subject's survival and recurrence data for up to five years after their surgery.

5 Study Drug

5.1 Description

The SGM-101 active ingredient is a covalent conjugate of the SGM-Ch511 anti-CEA chimeric monoclonal antibody with the fluorochrome BM-104 (Figure III.1-1). The BM-104 fluorochrome is conjugated to free amino groups of the antibody via an amide bond.

5.2 Treatment Regimen

The proposed dose of SGM-101 to be explored in this study is 5-10 mg to be administered intravenously over 30 minutes followed by a 50 mL flush of isotonic saline to account for the dead volume of the tubing. SGM-101 will be administered 3 to 5 days (+/-1 day) prior to surgery..

5.3 Method for Assigning Subjects to Treatment Groups

There will be only one treatment group for this trial.

5.4 Preparation and Administration of Study Drug

The SGM-101 active substance will present as a 5.0 mg/mL solution in 10 mM KH2PO4, 10 mM Na3 citrate, 300 mM arginine, 0.02% Tween-20, pH 6.0. The SGM-101 finished product is a sterile solution for injection of the same composition. It will be supplied as 4R type I amber glass vials capped with Flurotec© elastomeric stoppers and aluminum flip-off seals, containing a nominal volume of 2.3 mL and stored at +2 to +8°C. The SGM-101 active substance and investigational medicinal product (IMP) are manufactured and controlled in compliance with good manufacturing practice (GMP) by Polymun Immunobiologische Forschung (Klosterneuburg, Austria).

5.5 Subject Compliance Monitoring

Treatment includes a one-time dose of study drug with no additional compliance required after surgical exploration.

5.6 Prior and Concomitant Therapy

No prior or concomitant therapies will be collected or prohibited during this investigation.

5.7 Packaging

Carton label:

Study: SGM- CLIN03
SGM-101, Vial containing 2,3 mL (2ml extractable)
11,5mg (10 mg extractable)

SurgiMab
INTRAOPERATIVE FLUORESCENCE DIAGNOSTIC

Sterile solution for I.V. injection
See separate instructions for use
Store between +2°C and +8°C
Batch #: SGM1010618-A
Unit #:_______________
Expiry date:
Investigator name: ________________________________
Sponsor Address: 10 Parc Club du Millenaire – 1025 Avenue Henri Becquerel -34000 Montpellier - FRANCE
Tel : +33467798382

Caution: New Drug—Limited by Federal (or United States) law to investigational use

5.8 Blinding of Study Drug

As this is strictly an observational imaging study, no blinding of the drug will be necessary.

5.9 Receiving, Storage, Dispensing and Return

IDS will be responsible for all aspects of receiving, storage, dispensing and return of the drug. Drug will be shipped from the company manufacturing the drug directly to the IDS. Upon receipt of the drug an inventory will be performed and a drug receipt log filled out and signed. The shipment will be counted and verified that it contains all the items noted in the shipping invoice. Any discrepancies, damaged or unusable study drug in a given shipment will be documented in the study files.

6 Study Procedures

6.1 Visit 1- Initial Consult (up to 90 days before surgery)

Prior to the initiation of study-specific screening assessments the Investigator or a member of the study team must provide the subject(s) a complete explanation of the purpose and evaluations (procedures and assessments) of the study. Subsequently, the subject must sign and receive a copy of an Informed consent form to examine at their leisure. Once informed consent has been obtained, the eligibility of the subject

will be determined, and screening period assessments will be performed.

6.2 Visit 2- CHPS (infusion up to 5 days before surgery)

Visit two will consist of the infusion of SGM-101 and surgery. Based on the discretion of the surgeon, 25 mg of Benadryl may or may not be administered prior to infusion of study drug. Once the administration of the Benadryl, is completed, the drug will be administered by the surgeon or CHPS personnel. Note that SGM-101 should not be mixed with other medicinal products and should not be given simultaneously through the same intravenous line as another medicine. During this time, the subject will be monitored by CHPS personnel for signs of an allergic reaction. Vital signs (Heart rate, Blood Pressure, Pulse/Oxygen levels, body temperature and skin color) will first be collected and recorded at pre-infusion, next at 15-minute intervals after the infusion has begun up until the 45-minute infusion is complete (see chart below). This will include the 30 minutes for the infusion of the drug and the 15 minutes for the infusion of the flush.

	Pre-Infusion	Infusion Start	15 minutes after starting infusion	30 minutes after starting infusion; end of infusion	45 minutes after starting infusion	60 minutes after starting infusion
Time						
Temp						
HR						
BP						
O2 Sat						
Skin color						

6.3 Visit 3- Surgery

During the operation, imaging will take place before resection and after resection. Before resection, the surgeon will perform the standard of care operation in order to expose the tumor. Once the lung is exposed, the surgeon will use the intraoperative imaging device to videotape and discover if the tumor is fluorescent. Also, the surgeons will determine if there are any additional lesions that are fluorescent.

Once this is accomplished, we will proceed with the standard-of-care operation and resection. Then once the tumor is removed, the camera will be re-inserted to determine if there are any additional lesions still in the chest. If there is a lesion left behind, the surgeon will determine if this can be biopsied or not. If removing the additional lesion

would significantly alter the magnitude of the operation, then the nodule or residual cancer will not be removed. It can be monitored postoperatively by CAT scans. In this study, until we are confident of the false positive rate, no additional surgery will be performed based on imaging results alone. If the surgeon using his experience feels this additional location is cancer, then it will be biopsied and/or removed.

7 Statistical Plan

7.1 Sample Size Determination

Our analysis will be the following:

Data Analysis of a Diagnostic Test		Test result		Data Analysis
		True (accept H_0)	False (accept H_A)	
Disease present ("The Truth")	True (H_0)	TP (1 – β)	FN (type 2 error = β = 1-sens)	$sensitivity = \frac{TP}{TP + FN}$
	False (H_A)	FP (type 1 error = α = 1-spec)	TN (1 – α)	$specificity = \frac{TN}{TN + FP}$
Data Analysis		$PPV = \frac{TP}{TP + FP}$	$NPV = \frac{TN}{TN + FN}$	$Accuracy = efficiency = \frac{TP + TN}{TP + TN + FP + FN}$

True positive = fluorescent nodule, pathology is cancer
False positive = fluorescent nodule, pathology is no cancer
True negative = not fluorescent nodule, pathology is no cancer
False negative = not fluorescent nodule, pathology is cancer

Initially 10 subjects will be enrolled. We wish to be able to detect a false positive rate that is sufficiently high to warrant concern and trigger termination of enrollment pending review of results and procedures. If the first five or more subjects all have false positives (i.e., fluorescent nodules that are not cancer), we will terminate enrollment. This rule provides 87% power to detect a false positive rate of 30% or higher. If the true false positive rate is only 5%, the probability of early termination is 4%.

The sensitivity will be based on the single lung nodule that is being removed in a patient. Typically for lung cancer surgery, we are there to remove one nodule. That will be the basis of the calculations. Thus, the denominator will be 10 patients for the first Cohort, and another 10 patients for the second Cohort. (A total of 20 data points for the whole study). The calculations will be based on two pieces of data: tumor fluorescence and pathology. We will not be doing a detailed IHC study at this time.

If successful, we will consider 10 more subjects. Then, with 20 subjects, we will have 90% power to identify any unforeseen adverse event that occurs with a prevalence of at least 5%. We will use a Bayesian continuous safety monitoring design that registers a signal if the posterior probability that the chance of an adverse reaction is greater than 0.1 exceeds 80%. If we encounter a safety signal, we will suspend enrollment until the issue has been addressed.

The fraction of tumors on pathology that are identified by the imaging procedure will provide an estimate of the sensitivity of the procedure. Of the 20 enrolled subjects, we expect approximately 60%, or 12, to have fluorescent tumors. With 12 tumors, an exact 95% confidence interval for sensitivity would range from (0%, 12%) if no tumors are detected up to (88%, 100%) if all 12 are detected. We will compute estimates of the sensitivity (fraction of path-positive margins that are called correctly) and specificity (fraction of path-negative margins that are called correctly), and calculate 95% confidence intervals around them. A second aim will be to estimate the sensitivity of the procedure for identifying all lesions, including lesions that were not pre-identified. Because some subjects will contribute more than one lesion, we will estimate these parameters using random effects logistic regression models (in SAS Proc Glimmix) that allow for between-subject variability. These specimens will provide independent estimates of the positive and negative predictive values of discovering satellite nodules.

7.2 Statistical Methods

All data will be summarized, with means and standard deviations for continuous variables and proportions for categorical variables. Graphical techniques will be used for data exploration. Analyses will be primarily descriptive. The remainder of the statistical approaches is described above.

Null hypothesis = Lung cancers will not fluoresce with a CEA-targeted dye.
Alternative hypothesis = Lung cancers will fluoresce with a CEA-targeted dye.
Alpha = 0.25
Beta = 0.5

There are 20 patients, thus the denominator will be 20.
Each patient will contribute one nodule or cancer to the study.
Patients with more than one nodule typically do not get surgery because that represents metastatic disease and is considered non-surgical.

8 Safety and Adverse Events

8.1 Definitions

Unanticipated Problems Involving Risk to Subjects or Others

Any incident, experience, or outcome that meets all of the following criteria:

1. Unexpected in nature, severity, or frequency (i.e. not described in study-related documents such as the IRB-approved protocol or consent form, the investigators brochure, etc.)
2. Related or possibly related to participation in the research (i.e. possibly related means there is a reasonable possibility that the incident experience, or outcome may have been caused by the procedures involved in the research)

3. Suggests that the research places subjects or others at greater risk of harm (including physical, psychological, economic, or social harm).

All events that occur and meet the Common Terminology Criteria for Adverse Events (CTCAE version 4) published by the U.S. Department of Health and Human Services will be reported.

Adverse Event

An ***adverse event*** (AE) is any symptom, sign, illness or experience that develops or worsens in severity during the course of the study. Intercurrent illnesses or injuries should be regarded as adverse events. Abnormal results of diagnostic procedures are considered to be adverse events if the abnormality:

1. results in study withdrawal
2. is associated with a serious adverse event
3. is associated with clinical signs or symptoms
4. leads to additional treatment or to further diagnostic tests
5. is considered by the investigator to be of clinical significance

Serious Adverse Event

Adverse events are classified as serious or non-serious. A ***serious adverse event*** is any AE that is:

1. fatal
2. life-threatening
3. requires or prolongs hospital stay
4. results in persistent or significant disability or incapacity
5. a congenital anomaly or birth defect
6. an important medical event

Important medical events are those that may not be immediately life threatening, but are clearly of major clinical significance. They may jeopardize the subject, and may require intervention to prevent one of the other serious outcomes noted above. For example, drug overdose or abuse, a seizure that did not result in in-subject hospitalization or intensive treatment of bronchospasm in an emergency department would typically be considered serious. All adverse events that do not meet any of the criteria for serious should be regarded as non-serious adverse events.

Adverse Event Reporting Period

The study period during which adverse events must be reported is from the time the subject checks-in for SGM 101 study dye infusion visit until completion of subject's post-operative visit (approximately 2-4 weeks after visit 3 date of surgery). The following common surgical adverse events with a start date occurring after the subject's surgical procedure has begun will not be captured unless the adverse event remains a grade 4 at discharge: pain, Nausea, Vomiting, Fatigue, Inflammation, Swelling, Tenderness at the site of incision, Pneumothorax, Atelectasis, Hemothorax,

Hydropneumothorax, Drain placement for fluid retention and air leak, Excision site pain, Atrial Fibrillation, Renal Insufficiency, Constipation, Anemia, Sinus Bradycardia, Crepitus, Stress, Urinary Retention, Drowsy, Light headedness, Headache, Pneumonia, Hypotension, Hypertension, Frustration, Irritability , Restlessness, and Insomnia.

Preexisting Condition

A preexisting condition is one that is present at the start of the study. A preexisting condition should be recorded as an adverse event if the frequency, intensity, or the character of the condition worsens during the study period.

General Physical Examination Findings

At screening, any clinically significant abnormality should be recorded as a preexisting condition. At the end of the study, any new clinically significant findings/abnormalities that meet the definition of an adverse event must also be recorded and documented as an adverse event.

Post-study Adverse Event

All unresolved adverse events should be followed by the investigator until the events are resolved, the subject is lost to follow-up, or the adverse event is otherwise explained. At the last scheduled visit, the investigator should instruct each subject to report any subsequent event(s) that the subject, or the subject's personal physician, believes might reasonably be related to participation in this study.

Abnormal Laboratory Values

A clinical laboratory abnormality should be documented as an adverse event if any one of the following conditions is met:

1. The laboratory abnormality is not otherwise refuted by a repeat test to confirm the abnormality
2. The abnormality suggests a disease and/or organ toxicity
3. The abnormality is of a degree that requires active management; e.g. change of dose, discontinuation of the drug, more frequent follow-up assessments, further diagnostic investigation, etc.
4. The post-surgical laboratory abnormality falls outside the following ranges:
    a. Glucose: 50-500mg/dl
    b. Red Blood Cell: 2.15-12 MIl/uL
    c. Hemoglobin: 6.75-20.00g/dL
    d. Hematocrit: 20.0-60.0%
    e. Creatinine: 0.3-5.0mg/dL
    f. Sodium Level: 110-180mmol/L
    g. Potassium Level: 2.5-8.0mmol/L
    h. Chloride: 80-150 mmol/L
    i. CO_2: 10-60mmol/L
    j. Calcium Level: 6.0-20.0 mg/dL

V

5.

Hospitalization, Prolonged Hospitalization or Surgery

Any adverse event that results in hospitalization or prolonged hospitalization should be documented and reported as a serious adverse event unless specifically instructed otherwise in this protocol. Any condition responsible for surgery should be documented as an adverse event if the condition meets the criteria for and adverse event. Neither the condition, hospitalization, prolonged hospitalization, nor surgery are reported as an adverse event in the following circumstances:

1. Hospitalization or prolonged hospitalization for diagnostic or elective surgical procedures for a preexisting condition.
2. Surgery should not be reported as an outcome of an adverse event if the purpose of the surgery was elective or diagnostic and the outcome was uneventful.
3. Hospitalization or prolonged hospitalization required to allow efficacy measurement for the study.
4. Hospitalization or prolonged hospitalization for therapy of the target disease of the study, unless it is a worsening or increase in frequency of hospital admissions as judged by the clinical investigator.

8.2 Recording of Adverse Events

At each contact with the subject while on the study, the investigator must seek information on adverse events by specific questioning and, as appropriate, by examination. Information on all adverse events should be recorded immediately in the source document, and also in the appropriate adverse event module of the case report form (CRF). All clearly related signs, symptoms, and abnormal diagnostic procedures results should be recorded in the source document, though should be grouped under one diagnosis.

The clinical course of each adverse event should be followed until resolution, stabilization, or until it has been determined that the study treatment or participation is not the cause. Serious adverse events that are still ongoing at the end of the study period must be followed up to determine the final outcome. Any serious adverse event that occurs after the study period and is considered to be related to the study treatment or study participation should be recorded and reported immediately.

8.3 Reporting of Serious Adverse Events and Unanticipated Problems

Investigators must conform to the adverse event reporting timelines, formats and requirements of the UPenn IRB (8.3.1) and DSMC (8.4) to which are responsible, but at a minimum those events that must be reported are those that are:

1. related to study participation,
2. unexpected, and
3. serious or involve risks to subjects or others (see definitions, section 8.1).

If the report is supplied as a narrative, the minimum necessary information to be provided at the time of the initial report includes:

1. Study identifier
2. Study Center
3. Subject number
4. A description of the event
5. Date of onset
6. Current status
7. Whether study treatment was discontinued
8. The reason why the event is classified as serious
9. Investigator assessment of the association between the event and study treatment

Investigator reporting: notifying the Penn IRB

This section describes the requirements for safety reporting by investigators who are Penn faculty, affiliated with a Penn research site, or otherwise responsible for safety reporting to the Penn IRB. The University of Pennsylvania IRB (Penn IRB) requires expedited reporting of those events related to study participation that are unforeseen and indicate that participants or others are at increased risk of harm.

The Penn IRB will not acknowledge safety reports or bulk adverse event submissions that do not meet the criteria outlined below. The Penn IRB requires researchers to submit reports of the following problems within 10 working days from the time the investigator becomes aware of the event: Any adverse event (regardless of whether the event is serious or non-serious, on-site or off-site) that occurs any time during or after the research study, which in the opinion of the principal investigator is:

Unexpected (An event is “unexpected” when its specificity and severity are not accurately reflected in the protocol-related documents, such as the IRB-approved research protocol, any applicable investigator brochure, and the current IRB-approved informed consent document and other relevant sources of information, such as product labeling and package inserts.) AND Related to the research procedures (An event is “related to the research procedures” if in the opinion of the principal investigator, the event was more likely than not to be caused by the research procedures.

Investigator reporting: notifying the Pharmacovigilance company

All attributable SAEs will be reported to Vigipharm, the Pharmacovigilance company, that monitors the use of SGM-101.

Reporting Process

Unanticipated problems posing risks to subjects or others as noted above will be reported to the Penn IRB using the form: “Unanticipated Problems Posing Risks to

Subjects or Others Including Reportable Adverse Events" or as a written report of the event (including a description of the event with information regarding its fulfillment of the above criteria, follow-up/resolution and need for revision to consent form and/or other study documentation).

Copies of each report and documentation of IRB notification and receipt will be kept in the Clinical Investigator's study file.

Reporting Deaths: more rapid reporting requirements

Concerning deaths that occur during the course of a research study, the following describes the more rapid reporting requirement of the Penn IRB for specific situations:

- Report the event within 24 hours when the death is unforeseen (unexpected) and indicates participants or others are at increased risk of harm.
- Report the event within 72 hours, for all other deaths, regardless of whether the death is related to study participation.

For reportable deaths, the initial submission to the Penn IRB may be made by contacting the IRB Director or Associate Director. The AE/Unanticipated Problem Form is required as a follow up to the initial submission.

Other Reportable events:

For clinical drug trials, the following events are also reportable to the Penn IRB:

- Any adverse experience that, even without detailed analysis, represents a serious unexpected adverse event that is rare in the absence of drug exposure (such as agranulocytosis, hepatic necrosis, Stevens-Johnson syndrome).
- Any adverse event that would cause the investigator to modify the protocol or informed consent form, or would prompt other action by the IRB to assure protection of human subjects.
- Information that indicates a change to the risks or potential benefits of the research, in terms of severity or frequency. For example:
  - An interim analysis indicates that participants have a lower rate of response to treatment than initially expected.
  - Safety monitoring indicates that a particular side effect is more severe, or more frequent than initially expected.
  - A paper is published from another study that shows that an arm of your research study is of no therapeutic value.
- Change in FDA safety labeling or withdrawal from marketing of a drug, device, or biologic used in a research protocol.
- Breach of confidentiality
- Change to the protocol taken without prior IRB review to eliminate apparent immediate hazard to a
- research participant.
- Incarceration of a participant when the research was not previously approved under Subpart C and the investigator believes it is in the best interest of the subject to remain on the study.

- Complaint of a participant when the complaint indicates unexpected risks or the complaint cannot be resolved by the research team.
- Protocol violation (meaning an accidental or unintentional deviation from the IRB approved protocol) that in the opinion of the investigator placed one or more participants at increased risk, or affects the rights or welfare of subjects.

8.4 Reporting SAE's to the DSMC

All on-site SAEs for Penn subjects regardless of attribution or expectedness must be submitted to the DSMC within 10 days. Reports will be sent to the DSMC for **90 days** following the last date the subject received study treatment/therapy or was exposed to an investigational device.

All unexpected deaths or deaths related to the study agents/device must be reported within 24 hours. All other deaths should be reported within 30 days.

All adverse events which meet the following criteria will also be managed as described below:

All Grade 3 or higher events will be reported to the DSMC within 10 days of knowledge of the event. All unexpected deaths will be reported within 24 hours of knowledge of the event.

All other deaths will be reported within 30 days of knowledge of the event.

In addition, any adverse events will be recorded promptly in CTMS (clinical research management database).

In addition, any adverse events will be recorded in CTMS (clinical research management database).

8.5 Unblinding Procedures

As this is an open-label study, there is no need to specify unblinding procedures.

8.6 Stopping Rules

Should there be any evidence that arises that this is not helpful in subjects or early given various true toxicity rate, we will early terminate this protocol

8.7 Medical Monitoring

It is the responsibility of the Principal Investigator to oversee the safety of the study at his site. This safety monitoring will include careful assessment and appropriate reporting of adverse events as noted above, as well as the construction and implementation of a site data and safety-monitoring plan (see section 10 Auditing, Monitoring and Inspecting). Medical monitoring will include a regular assessment of the number and type of serious adverse events.

9 Data Handling and Record Keeping

9.1 Confidentiality

Information about study subjects will be kept confidential and managed according to the requirements of the Health Insurance Portability and Accountability Act of 1996 (HIPAA). Those regulations require a signed subject authorization informing the subject of the following:

- What protected health information (PHI) will be collected from subjects in this study
- Who will have access to that information and why
- Who will use or disclose that information
- The rights of a research subject to revoke their authorization for use of their PHI.

In the event that a subject revokes authorization to collect or use PHI, the investigator, by regulation, retains the ability to use all information collected prior to the revocation of subject authorization. For subjects that have revoked authorization to collect or use PHI, attempts should be made to obtain permission to collect at least vital status (i.e. that the subject is alive) at the end of their scheduled study period.

9.2 Source Documents

Source data is all information, original records of clinical findings, observations, or other activities in a clinical trial necessary for the reconstruction and evaluation of the trial. Source data are contained in source documents. Examples of these original documents, and data records include: hospital records, clinical and office charts, laboratory notes, memoranda, subjects' diaries or evaluation checklists, pharmacy dispensing records, recorded data from automated instruments, copies or transcriptions certified after verification as being accurate and complete, microfiches, photographic negatives, microfilm or magnetic media, x-rays, subject files, and records kept at the pharmacy, at the laboratories, and at medico-technical departments involved in the clinical trial.

9.3 Case Report Forms

The study case report form (CRF) is the primary data collection instrument for the study. All data requested on the CRF must be recorded. All missing data must be explained. If a space on the CRF is left blank because the procedure was not done or the question was not asked, write "N/D". If the item is not applicable to the individual case, write "N/A". All entries should be printed legibly in black ink. If any entry error has been made, to correct such an error, draw a single straight line through the incorrect entry and enter the correct data above it. All such changes must be initialed and dated. DO NOT ERASE OR WHITE OUT ERRORS. For clarification of illegible or uncertain entries, print the clarification above the item, then initial and date it.

9.4 Records Retention

It is the investigator's responsibility to retain study essential documents for at least 2 years. The investigator will continue to report to the Institutional Review Board and the Cancer Trials Scientific Review Monitoring Committee during this period. The study will be review by the CTSRMC every six months.

PennCTMS, a clinical trial management system, will be used to register subject information as a participant in the study and to allow the research data to be entered and stored for the purpose of data analysis and any other required activity for the purpose of the conduct of the research.

REDcap, a secure web application, used for building and managing online surveys and databases, will be used to store and record subject surgical and demographic data for post-operative teaching and review for the research staff.

10 Study Monitoring, Auditing, and Inspecting

10.1 Study Monitoring Plan

This study will be monitored according to the monitoring plan in Attachment 5. The investigator will allocate adequate time for such monitoring activities. The Investigator will also ensure that the monitor or other compliance or quality assurance reviewer is given access to all the above noted study-related documents and study related facilities (e.g. pharmacy, diagnostic laboratory, etc.), and has adequate space to conduct the monitoring visit. The study will be audited by the Department of Compliance and Monitoring (DOCM) on behalf of the DSMC in accordance with the NCI approved Institutional Data Safety and Monitoring Plan (DSMP).

10.2 Auditing and Inspecting

The investigator will permit study-related monitoring, audits, and inspections by the EC/IRB, government regulatory bodies, and University compliance and quality assurance groups of all study related documents (e.g. source documents, regulatory documents, data collection instruments, study data etc.). The investigator will ensure the capability for inspections of applicable study-related facilities (e.g. pharmacy, diagnostic laboratory, etc.).

Participation as an investigator in this study implies acceptance of potential inspection by government regulatory authorities and applicable University compliance and quality assurance offices.

10.3 Reporting of Exceptions and Deviations

Exception

A one-time, **intentional** action or process that departs from the IRB and CTSRMC approved study protocol, intended for **one** occurrence. If the action disrupts the

study progress, such that the study design or outcome (endpoints) may be compromised, or the action compromises the safety and welfare of study subjects, **advance** documented IRB and DSMC approval is required.

- For exceptions on Industry or Cooperative group sponsored protocols, written approval must be obtained from the Sponsor prior to submitting your exception request to the DSMC.
- For in-house studies with a Medical Monitor or Safety Monitoring Committee (not DSMB), approval must be obtained from the Medical Monitor or Safety Monitoring Committee prior to submitting your exception request to the DSMC.

Deviation

A one-time, **unintentional** action or process that departs from the IRB and CTSRMC approved study protocol, involving one incident and **identified retrospectively**, after the event occurred. If the impact on the protocol disrupts the study design, may affect the outcome (endpoints) or compromises the safety and welfare of the subjects, the deviation must be reported to the CTSRMC within 5 business days and the IRB within 10 business days.

Examples of Exceptions/Deviations that must be submitted (not meant to be inclusive)

May/can/have affects/affected subject safety. So, a subject missing a visit is not an issue unless a critical/important treatment or procedure was missed and must have been done at that specific time.

- Violate eligibility
- Dose adjustment
- Stopping criteria
- Affect sample size (adding more subjects, decreasing number of subjects, changing the number of subject in a specific arm/cohort)

11 Ethical Considerations

This study is to be conducted according to US and international standards of Good Clinical Practice (FDA Title 21 part 312 and International Conference on Harmonization guidelines), applicable government regulations and Institutional research policies and procedures.

This protocol and any amendments will be submitted to a properly constituted independent Ethics Committee (EC) or Institutional Review Board (IRB), in agreement with local legal prescriptions, for formal approval of the study conduct. The decision of the EC/IRB concerning the conduct of the study will be made in writing to the investigator before commencement of this study.

All subjects for this study will be provided a consent form describing this study and providing sufficient information for subjects to make an informed decision about their participation in this study. See Attachment 1 for a copy of the Subject Informed Consent Form. This consent form will be submitted with the protocol for review and approval by the EC/IRB for the study. The formal consent of a subject, using the EC/IRB-approved consent form, must be obtained before that subject undergoes any study procedure. The consent form must be signed by the subject or legally acceptable surrogate, and the investigator-designated research professional obtaining the consent.

12 Study Finances

12.1 Funding Source

This study will be funded by the Center for Precision Surgery in the Abramson Cancer Center which does not come from the makers of the probe used in the study.

12.2 Conflict of Interest

The Investigator states that no conflict of interest exists within this study

13 Publication Plan

No part of this study will be published without conforming to the regulations of the Department of Surgery of the University of Pennsylvania standards for publishing.

14 Attachments

1. The proposed consent form with modified HIPAA
2. Data monitoring plan
3. Adverse Event log
4. Nurse Flow Sheet
5. Eligibility Criteria
6. Consent process
7. Surgery confirmation

15 References

1. Siegel, R. L., Miller, K. D., and Jemal, A. Cancer Statistics, 2017. CA Cancer J Clin, *67:* 7-30, 2017.
2. Kennedy, G. T., Newton, A., Predina, J., and Singhal, S. Intraoperative near-infrared imaging of mesothelioma. Transl Lung Cancer Res, *6:* 279-284, 2017.
3. Newton, A. D., Kennedy, G. T., Predina, J. D., Low, P. S., and Singhal, S. Intraoperative molecular imaging to identify lung adenocarcinomas. Journal of Thoracic Disease, *8:* S697, 2016.

4. Newton, A. D., Predina, J. D., Nie, S., Low, P. S., and Singhal, S. Intraoperative fluorescence imaging in thoracic surgery. J Surg Oncol, 2018.
5. Predina, J. D., Fedor, D., Newton, A. D., Xia, L., Lee, J. Y. K., Guzzo, T., Drebin, J., and Singhal, S. Intraoperative Molecular Imaging: The Surgical Oncologist's North Star. Ann Surg, 2017.
6. Baisi, A., Raveglia, F., De Simone, M., and Cioffi, U. Do Tumor Size and Carcinoembryonic Antigen Level Affect Surgical Management of Partially Solid Early-Stage Lung Cancer? Ann Thorac Surg, *103:* 1036, 2017.
7. Chen, X., Wang, X., He, H., Liu, Z., Hu, J. F., and Li, W. Combination of circulating tumor cells with serum carcinoembryonic antigen enhances clinical prediction of non-small cell lung cancer. PLoS ONE, *10:* e0126276, 2015.
8. Grunnet, M. and Sorensen, J. B. Carcinoembryonic antigen (CEA) as tumor marker in lung cancer. Lung Cancer, *76:* 138-143, 2012.
9. Kuo, S. W., Chen, J. S., Huang, P. M., Hsu, H. H., Lai, H. S., and Lee, J. M. Prognostic significance of histologic differentiation, carcinoembryonic antigen value, and lymphovascular invasion in stage I non-small cell lung cancer. J Thorac Cardiovasc Surg, *148:* 1200-1207 e1203, 2014.
10. Triphuridet, N., Vidhyarkorn, S., Worakitsitisatorn, A., Sricharunrat, T., Teerayathanakul, N., Auewarakul, C., Chungklay, N., Krongthong, W., Luengingkasoot, S., Sornsamdang, G., Patumanond, J., and Sritipsukho, P. Screening values of carcinoembryonic antigen and cytokeratin 19 fragment for lung cancer in combination with low-dose computed tomography in high-risk populations: Initial and 2-year screening outcomes. Lung Cancer, *122:* 243-248, 2018.
11. Holdenrieder, S., Wehnl, B., Hettwer, K., Simon, K., Uhlig, S., and Dayyani, F. Carcinoembryonic antigen and cytokeratin-19 fragments for assessment of therapy response in non-small cell lung cancer: a systematic review and meta-analysis. Br J Cancer, *116:* 1037-1045, 2017.
12. Zhao, X. M., Zhao, J., Xing, K. L., Sun, S., Luo, Z. G., Wang, H. J., Wang, J. L., Chang, J. H., and Wu, X. H. Prognostic and predictive value of serum carcinoembryonic antigen levels in advanced non-small cell lung cancer patients with epidermal growth factor receptor sensitive mutations and receiving tyrosine kinase inhibitors. Oncotarget, *8:* 70865-70873, 2017.
13. Zhang, L., Liu, D., Li, L., Pu, D., Zhou, P., Jing, Y., Yu, H., Wang, Y., Zhu, Y., He, Y., Li, Y., Zhao, S., Qiu, Z., and Li, W. The important role of circulating CYFRA21-1 in metastasis diagnosis and prognostic value compared with carcinoembryonic antigen and neuron-specific enolase in lung cancer patients. BMC Cancer, *17:* 96, 2017.
14. Goldenberg, D. M., DeLand, F., Kim, E., Bennett, S., Primus, F. J., van Nagell, J. R., Jr., Estes, N., DeSimone, P., and Rayburn, P. Use of radiolabeled antibodies to carcinoembryonic antigen for the detection and localization of diverse cancers by external photoscanning. N Engl J Med, *298:* 1384-1386, 1978.
15. Mach, J. P., Carrel, S., Forni, M., Ritschard, J., Donath, A., and Alberto, P. Tumor localization of radiolabeled antibodies against carcinoembryonic antigen in patients with carcinoma: a critical evaluation. N Engl J Med, *303:* 5-10, 1980.

16. Folli, S., Wagnieres, G., Pelegrin, A., Calmes, J. M., Braichotte, D., Buchegger, F., Chalandon, Y., Hardman, N., Heusser, C., Givel, J. C., and et al. Immunophotodiagnosis of colon carcinomas in patients injected with fluoresceinated chimeric antibodies against carcinoembryonic antigen. Proc Natl Acad Sci U S A, *89:* 7973-7977, 1992.
17. Gutowski, M., Carcenac, M., Pourquier, D., Larroque, C., Saint-Aubert, B., Rouanet, P., and Pelegrin, A. Intraoperative immunophotodetection for radical resection of cancers: evaluation in an experimental model. Clin Cancer Res, *7:* 1142-1148, 2001.
18. Boonstra, M. C., Tolner, B., Schaafsma, B. E., Boogerd, L. S., Prevoo, H. A., Bhavsar, G., Kuppen, P. J., Sier, C. F., Bonsing, B. A., Frangioni, J. V., van de Velde, C. J., Chester, K. A., and Vahrmeijer, A. L. Preclinical evaluation of a novel CEA-targeting near-infrared fluorescent tracer delineating colorectal and pancreatic tumors. Int J Cancer, *137:* 1910-1920, 2015.
19. Metildi, C. A., Kaushal, S., Snyder, C. S., Hoffman, R. M., and Bouvet, M. Fluorescence-guided surgery of human colon cancer increases complete resection resulting in cures in an orthotopic nude mouse model. J Surg Res, *179:* 87-93, 2013.
20. Metildi, C. A., Kaushal, S., Pu, M., Messer, K. A., Luiken, G. A., Moossa, A. R., Hoffman, R. M., and Bouvet, M. Fluorescence-guided surgery with a fluorophore-conjugated antibody to carcinoembryonic antigen (CEA), that highlights the tumor, improves surgical resection and increases survival in orthotopic mouse models of human pancreatic cancer. Ann Surg Oncol, *21:* 1405-1411, 2014.
21. Gutowski, M., Framery, B., Boonstra, M. C., Garambois, V., Quenet, F., Dumas, K., Scherninski, F., Cailler, F., Vahrmeijer, A. L., and Pelegrin, A. SGM-101: An innovative near-infrared dye-antibody conjugate that targets CEA for fluorescence-guided surgery. Surg Oncol, *26:* 153-162, 2017.
