# Supplementary material for: Carcinoembryonic Antigen-Related Cell Adhesion Molecule Type 5 Receptor-Targeted Fluorescent Intraoperative Molecular Imaging Tracer for Lung Cancer: A Nonrandomized Controlled Trial
Source: JAMA Netw Open. Author manuscript; Available in PMC 2024 Jan 3. (PMC10292762; doi:10.1001/jamanetworkopen.2022.52885)
Supplement: Supp 2 — eMethods. Supplemental Methods eTable 1. Fisher’s Exact Test Exploring CEACAM5 Expression With Respect to Patient/Histopathologic Characteristics in 33 Patients Who Underwent Resection of Lung Adenocarcinoma eTable 2. Demographic and Histopathologic Characteristics of the 10 Patients Included in the Study eTable 3. List of Complications Observed in the Patient Cohort for the Study eTable 4. Histopathologic Details of Patients in the Control Group With Corresponding SGM-101-Related Intraoperative Findings eTable 5. Primary Malignancy Surgical Characteristics of the Patients in the Metastasis Control Group eTable 6. Characteristics of SGM-101 Guided Resections of the Lung Nodules in the Cohort eFigure 1. CEACAM5 Core Biopsy IHC Staining of 33 Consecutive Lung Adenocarcinoma Patients With (1) Cancer Tissue, (2) Cancer Adjacent Normal, and (3) Distant Normal Lung Demonstrating 22/33 Positive CEACAM5 Presence eFigure 2. Higher-Stage Lung Adenocarcinoma Correlated With an Increased Rate of CEACAM5 Expression (AJCC 8th ed) eFigure 3. TCGA Database Analysis Demonstrates CEACAM5 Expression in Various Prevalent Solid Organ Malignancies, With Colorectal Adenocarcinoma Demonstrating the Highest mRNA Expression (16 Log2(Value +1)) and Melanoma Demonstrating the Lowest Median Expression (1 (Log2(Value +1)) eFigure 4. Patients 3 and 4 With Normal Serum CEA Levels With SGM-101 Ex Vivo Localization of Subcentimeter Nodules eFigure 5. SGM-101 Fluorescence Correlates With CEACAM5 Presence by IHC (Patient 3) eFigure 6. SGM-101 Localization to CEACAM5+ Areas eFigure 7. CEACAM5 IHC and Serum CEACAM5/CEA Can Predict SGM-101 Fluorescence Success and Tumor Localization eFigure 8. Intraoperative Findings for Patient 2 in the Metastatic Control Cohort During SGM-101 Guided Lung Nodule Resection eFigure 9. Overview of CEACAM5-Targeted Fluorescence Localization of Lung Nodules With SGM-101 [file NIHMS1901881-supplement-Supp_2.pdf]

Supplementary Online Content

Azari F, Meijer RPJ, Kennedy GT, et al. Carcinoembryonic antigen–related cell adhesion molecule type 5 receptor–targeted fluorescent intraoperative molecular imaging tracer for lung cancer: a nonrandomized controlled trial. *JAMA Netw Open*. 2023;6(1):e2252885. doi:10.1001/jamanetworkopen.2022.52885

eMethods. Supplemental Methods
eTable 1. Fisher's Exact Test Exploring CEACAM5 Expression With Respect to Patient/Histopathologic Characteristics in 33 Patients Who Underwent Resection of Lung Adenocarcinoma
eTable 2. Demographic and Histopathologic Characteristics of the 10 Patients Included in the Study
eTable 3. List of Complications Observed in the Patient Cohort for the Study
eTable 4. Histopathologic Details of Patients in the Control Group With Corresponding SGM-101-Related Intraoperative Findings
eTable 5. Primary Malignancy Surgical Characteristics of the Patients in the Metastasis Control Group
eTable 6. Characteristics of SGM-101 Guided Resections of the Lung Nodules in the Cohort
eFigure 1. CEACAM5 Core Biopsy IHC Staining of 33 Consecutive Lung Adenocarcinoma Patients With (1) Cancer Tissue, (2) Cancer Adjacent Normal, and (3) Distant Normal Lung Demonstrating 22/33 Positive CEACAM5 Presence
eFigure 2. Higher-Stage Lung Adenocarcinoma Correlated With an Increased Rate of CEACAM5 Expression (AJCC 8th ed)
eFigure 3. TCGA Database Analysis Demonstrates CEACAM5 Expression in Various Prevalent Solid Organ Malignancies, With Colorectal Adenocarcinoma Demonstrating the Highest mRNA Expression (16 Log2(Value+1)) and Melanoma Demonstrating the Lowest Median Expression (1 (Log2(Value+1))
eFigure 4. Patients 3 and 4 With Normal Serum CEA Levels With SGM-101 Ex Vivo Localization of Subcentimeter Nodules
eFigure 5. SGM-101 Fluorescence Correlates With CEACAM5 Presence by IHC (Patient 3)
eFigure 6. SGM-101 Localization to CEACAM5+ Areas
eFigure 7. CEACAM5 IHC and Serum CEACAM5/CEA Can Predict SGM-101 Fluorescence Success and Tumor Localization
eFigure 8. Intraoperative Findings for Patient 2 in the Metastatic Control Cohort During SGM-101 Guided Lung Nodule Resection
eFigure 9. Overview of CEACAM5-Targeted Fluorescence Localization of Lung Nodules With SGM-101

This supplementary material has been provided by the authors to give readers additional information about their work.

eMethods. Supplemental Methods

Assessing CEACAM5 expression in human lung adenocarcinoma core biopsies

*Immunohistochemistry**:***

Under a University of Pennsylvania Institutional Review Board-approved protocol, histologic specimens of tumor, adjacent normal margin, and distal normal parenchyma from 33 consecutive lung adenocarcinoma patients were obtained from the Hospital University of Pennsylvania's Biobank. Samples were prepared and immunostained at the pathology core for CEA (CEACAM5) using anti-CEA antibody, thyroid transcription factor 1 (TTF-1), CK 5/6, p63, and Ki-67. Once stained, a certified pathologist manually scored the specimens using an established scoring system ranging from 0 to 3+. The tumor was considered positive when more than 10% of malignant cells were positively stained. Overexpression of CEACAM5 was defined as a score of 2+ or 3+, and mild expression of CEACAM5 was defined as a score of 1+.

TCGA and cBioPortal CEACAM5 Expression Primary Tumor-Derived Data Set Analysis:

Messenger RNA (mRNA) expression data of CEACAM5 in human cell lines generated by RNA-seq analysis were downloaded from the GDC data portal (TCGA), CCLE, and cBioPortal on 1/1/2022 and sorted by primary tumor "type". Samples with no data or no CEACAM5 expression were excluded. CEACAM5 expression was then compared to normal human tissues using the cBioPortal Integrative Analysis Platform[28].

© 2023 Azari F et al. *JAMA Network Open.*

Inclusion/Exclusion Criteria:

Overall Inclusion Criteria:

Patients aged 18 years or older with suspicion of gastrointestinal pulmonary metastasis or primary lung malignancy who were scheduled for either wedge pulmonary metastasectomy: metastasis arm or anatomic segmentectomy/lobectomy/pneumonectomy via video-assisted thoracoscopic resection (VATS), thoracotomy, or median sternotomy were included in the study. Patients were screened and evaluated for pulmonary nodules with cross-sectional imaging, including fine-cut 1-mm thickness respiratory gated CT scanning and PET-CT. Patients with suspicion of pulmonary metastases were evaluated according to oncologic guidelines, including abdominal CT, PET-CT, colonoscopy (colorectal) and MRI (pancreaticobiliary primary). All patients had serum CEA levels assessed pre- and postoperatively regardless of diagnosis. Patients with multivisceral disseminated disease who would not benefit from pulmonary metastasectomy were excluded from the study. A negative serum pregnancy test at screening followed by a negative urine pregnancy test on the day of surgery or day of admission for female patients of childbearing potential, female patients of childbearing potential or less than 2 years postmenopausal agree to use an acceptable form of contraception from the time of signing informed consent until 30 days after study completion, ability to understand the requirements of the study, provide written informed consent and authorization of use and disclosure of protected health information, and agree to abide by the study restrictions and to return for the required assessments.

Overall Exclusion Criteria:

Exclusion criteria for enrollment in the studies included previous exposure to fluorescent tracers, any medical condition that in the opinion of the investigators could potentially jeopardize the safety of the patient, history of anaphylactic reactions or severe allergies, history of allergy to

© 2023 Azari F et al. *JAMA Network Open.*

any of the components of SGM-101 or CEA targeted antibodies, pregnancy, positive pregnancy test, clinically significant abnormalities on electrocardiogram (ECG) at screening, presence of any psychological, familial, sociological or geographical condition potentially hampering compliance with the study protocol and follow-up schedule, impaired renal function defined as estimated glomerular filtration rate (eGFR) < 50 mL/min/1.73 m2, impaired liver function defined as values > 3x the upper limit of normal for alanine aminotransferase (ALT) or aspartate aminotransferase (AST), alkaline phosphatase (ALP), or total bilirubin, severely altered hematologic parameters including thrombocytopenia (PLT count <50,000/uL), neutropenia (ANC<1500), Hgb<7.0(gm/dL). Additional nonlaboratory exclusion criteria included patients who received an investigational agent in another investigational drug or vaccine trial within 30 days prior to surgery, had known sensitivity to fluorescent light, underwent chemoradiotherapy within 6 weeks of surgical intervention, had a Karnofsky performance status less than 70%, had a median ASA class 4 or higher, had other preexisting terminal diagnoses, and had an ejection fraction lower than 30%.

SGM-101 Parameters:

The BM-104 fluorochrome is conjugated to free amino groups of the antibody via an amide bond. The SGM-101 drug substance presents as a mixture of fluorochrome-antibody conjugate species of different conjugation levels, ranging from 1 to 6 BM104 molecules per antibody and of unconjugated species. The human constant domains of the antibody are of the G1m3 allotype for the heavy chains and Km3 for the light chains. The SGM-Ch511-BM-104 conjugates are characterized by absorbance peaks at 280 nm (antibody) and 685 nm (fluorochrome). The excitation and emission wavelength peaks of SGM-101 are 685 and 704 nm, respectively.

© 2023 Azari F et al. *JAMA Network Open.*

Camera Systems

In situ and ex vivo, fluorescent imaging was performed using the Artemis and Spectrum Handheld Camera System, open-field and laparoscopic, manufactured by Quest Medical Imaging (The Netherlands). The camera system consists of three wavelength-isolated light sources, including a “white” light source and two separate NIR light sources. For this study, the Cy5,5 filter setting (fluorescent range, 680 ± 30 nm) was used. Color video and fluorescence images were acquired simultaneously by separate sensors and displayed in real time using custom-built optics and software, thereby displaying color video and NIR fluorescence images separately. A pseudocolored (lime green) merged image of the color video and fluorescence images was also generated. The gain and exposure time settings were controlled using Quest software. An average gain setting of 25 was used, and the exposure time was varied between 60 and 120 ms according to the clinical situation. The camera was attached to a freely moveable arm. During surgery, the camera and moveable arm were enclosed in a sterile shield and drape (Medical Technique Inc., Tucson, AZ, USA)[29].

Outcomes:

The primary study objectives were to assess the sensitivity and specificity of SGM-101 in the detection of CEACAM5+ lung nodules during surgery. The secondary outcome of the study was the assessment of concordance between fluorescence intensity and final pathologic diagnosis.

The safety of the study will be determined via incidence rates of all AEs and treatment-emergent AEs (TEAEs) from the time of SGM-101 administration through follow-up using the National Cancer Institute Common Terminology Criteria for Adverse Events.

© 2023 Azari F et al. *JAMA Network Open.*

Clinical Feasibility of CEACAM5 Targeting in NSCLC

We initially wanted to explore the clinical feasibility of targeting CEACAM5 with SGM-101 in our cohort of patients diagnosed with lung adenocarcinoma. CEACAM5 expression and correlation with SGM-101 fluorescence have been previously performed in GI malignancies, but no data are available for NSCLC. Thirty-three consecutive patients with a diagnosis of lung adenocarcinoma underwent core biopsies of the tumor, tumor adjacent margin, and normal lung by dedicated thoracic pathologists. A total of 22/33 patients had positive CEACAM5 staining (4 (1+), 11 (2+), 7 (3+)). No 2+ or 3+ staining was observed for margins or normal parenchyma (S-1, Table 1). Assessment of biopsy samples demonstrated a correlation of advanced stage disease with an increased CEACAM5 IHC staining score (S-2). Demographics and histopathologic details are given in Supplementary Table 1 and Supplementary Figure 1.

© 2023 Azari F et al. *JAMA Network Open.*

eTable 1. Fisher's Exact Test Exploring CEACAM5 Expression With Respect to Patient/Histopathologic Characteristics In 33 Patients Who Underwent Resection of Lung Adenocarcinoma

**Variable**	**CEACAM5 Staining by Immunohistochemistry**		
	**Tumor Cells**		
	**Negative**	**Positive**	
**Total** (n, %)	11 (33)	22 (67)	
**Gender**			
Male (n=10)	3 (27)	7 (32)	*p*=0.441
Female (n=23)	8 (73)	15 (68)	
**Age**			
≤65 (n=14)	6 (55)	8 (36)	*P*<0.05
≥66 (n=19)	5 (45)	14 (64)	
**CEACAM5 Margin Positivity**			
Yes (n=4)	0 (0)	4 (18)	*P*<0.05
No (n=29)	11 (100)	18 (82)	
**Race**			
White (n=14)	4 (36)	10 (46)	*P*<0.05
Black (n=7)	3 (28)	4 (18)	
Asian (n=8)	2 (18)	6 (27)	
Unknown (4)	2 (18)	2 (9)	
**CEACAM5 Expression Adjacent Normal Tissue**			
No (n=32)	11 (100)	21 (95)	*P<0.05*
Yes (n=1)	0 (0)	1 (5)	

Majority of patients were females, older, and identified as white. CEACAM5 was minimally detected on margin assessment and there was minimal expression in normal parenchyma. Values are presented as n (%) of patients in respective rows unless otherwise indicated.

*Statistically significant association

© 2023 Azari F et al. *JAMA Network Open.*

**eTable 2**. Demographic and Histopathologic Characteristics of the 10 Patients Included in the Study	
**Patient Characteristics**	
	Number (%) or Median [IQR]
Total Patients Enrolled	10
Study Arm Patient (n)	
Metastasis (M)	5 (50%)
Primary Lung Nodule (L)	5 (50%)
Age (years)	66 [58-69]
Sex	
Male	5 (50%) (M=3, L=2)
Female	5 (50%) (M=2, L=3)
Race	
White	6 (60%) (M=2, L=4)
Black	1 (10%) (M=1, L=0)
Asian	0 (0%)
Other/Unknown	3 (30%) (M=2, L=1)
Any Smoking History	8 (80%) (M=3, L=5)
Pack Years	32.7 [10–38.9]
Time from Infusion to Resection (hrs)	92.88 [91.2-94.67]
SGM-101 Dose (mg)	10 (M=10 mg, L=10 mg)
Serum CEA Level (ng/mL)	3.0 [2-3.5]
Median ASA Classification	3 (M=3, L=3)
Hospital Length of Stay (days)	1.96 (1.5-4)
30 Day Mortality	0
90 Day Mortality	0
**Lesion Characteristics**	
Total Lesions	14 (M=7, L=7)
Size of Lesion (cm)	0.91 [0.9-2]
PET SUV	3.9 [1.8-5.7]
Tumor Location	

© 2023 Azari F et al. *JAMA Network Open.*

RUL	5 (35%) (M=2, L=3)
RML	3 (22%) (M=1, L=2)
RLL	4 (28%) (M=3, L=1)
LUL	2 (15%) (M=1, L=1)
LLL	0
Pleura	0
Chest Wall	0
Final Pathology	
Primary Lung Nodule Cohort	7 (50%)
Primary Lung Cancer	3 (42%)
Invasive Adenocarcinoma	1 (14%)
Minimally Invasive Adenocarcinoma	2 (28%)
Adenocarcinoma *in situ*	0
Squamous Cell Carcinoma	0
Small Cell Lung Cancer	0
Carcinoid Tumor	0
Benign Lesion	1 (14%)
Pulmonary Metastasis Cohort	7 (50%)
Colorectal Metastasis	4 (57%)
Pancreaticobiliary Metastasis	1 (14%)
Benign	2 (28%)
Tumor Differentiation (n=11)	
Well Differentiated	6 (42%) (M=1, L=5)
Moderately Differentiated	1 (29%) (M=0 , L=1)
Poorly Differentiated	4(29%) (M=4, L=0)

Of note, 1 patient in the metastasis group enrolled and completed infusion but did not complete surgery to ECG changes in the preoperative setting.

© 2023 Azari F et al. *JAMA Network Open.*

eTable 3. List of Complications Observed in the Patient Cohort for the Study

**Patient #**	**Complication Organ Class**	**Complication Type**	**Clavien–Dindo Class**	**Setting**	**SGM-101 Infusion Related**
1	Cardiac	Atrial fibrillation	II	Post-Operative	No
2	Musculoskeletal	Incisional Pain	II	Post-Operative	No
3	None	None	N/A	N/A	N/A
4	Genitourinary	Urinary Retention	I	Post-Operative	No
5	Cardiac	Abnormal Stress Test	IIIb	Pre-Operative	No
6	Musculoskeletal	Incisional Pain	II	Post-Operative	No
7	None	None	N/A	N/A	N/A
8	Cardiac	Atrial fibrillation	II	Post-Operative	No
9	None	None	N/A	N/A	N/A
10	Respiratory	Prolonged Air Leak	I	Post-Operative	No

© 2023 Azari F et al. *JAMA Network Open.*

eTable 4. Histopathologic Details of Patients in the Control Group With Corresponding SGM-101-Related Intraoperative Findings

Patient #	Final Pathology	CEACAM5 IHC Score	SGM-101 Fluorescence	Serum CEA Elevated	Serum CEA Normalization Post Resection
1	Adenocarcinoma of the Colon	3+	Yes	Yes	Yes
2	Adenocarcinoma of the Colon	3+	Yes	Yes	Yes
3	Pancreatic Adenocarcinoma	1+	Fluorescence Microscopy Only	No	N/A
4	Colorectal Adenocarcinoma	2+	Fluorescence Microscopy Only	No	N/A
5	N/A	N/A	N/A	Yes	N/A

© 2023 Azari F et al. *JAMA Network Open.*

eTable 5. Primary Malignancy Surgical Characteristics of the Patients in the Metastasis Control Group

Patient #	Primary Malignancy Origin	Index Operation	Neo-Adjuvant Therapy	Adjuvant Therapy	Serum CEA Elevated
1	Colorectal	Sigmoidectomy	No	Yes	Yes
2	Colorectal	Right Hemicolectomy	No	No	Yes
3	Pancreatic	Pylorus Preserving Pancreaticoduodenectomy	Yes	Yes	No
4	Colorectal	Low Anterior Resection	Yes	No	No
5	Colorectal	Extended Right Hemicolectomy	No	No	Yes

© 2023 Azari F et al. *JAMA Network Open.*

eTable 6. Characteristics of SGM-101 Guided Resections of the Lung Nodules in the Cohort

**Patient #**	**Final Pathology**	**CEACAM5 IHC Score**	**SGM-101 Fluorescence**	**Serum CEA Normalized Post Resection**
6	Benign	0	No (Figure 5)	N/A
7	Minimally Invasive Adenocarcinoma	1+	Ex-Vivo Only	N/A
8	Invasive Adenocarcinoma	2+	Yes (Figure 5)	N/A
9	Benign	0	No	N/A
10	Minimally Invasive Adenocarcinoma	0	No	N/A

© 2023 Azari F et al. *JAMA Network Open.*

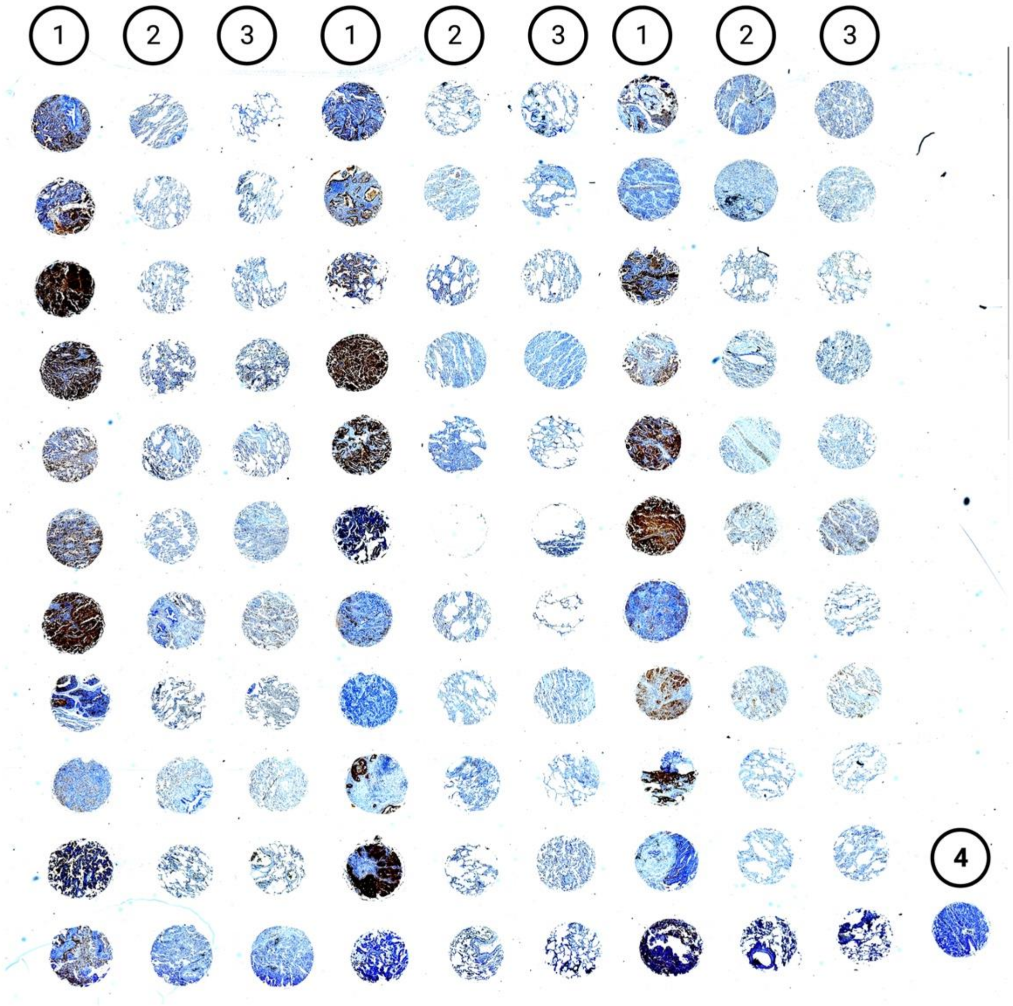

eFigure 1. CEACAM5 Core Biopsy IHC Staining of 33 Consecutive Lung Adenocarcinoma Patients With (1) Cancer Tissue, (2) Cancer Adjacent Normal, and (3) Distant Normal Lung Demonstrating 22/33 Positive CEACAM5 Presence
Representative images of CEACAM5+ and CEACAM5- core biopsy samples with CEACAM5+ presence noted with chromogenic IHC stain (brown) and counterstain with hematoxylin (blue). The absence of chromogenic CEACAM5 staining indicates the absence of glycoprotein expression.

© 2023 Azari F et al. *JAMA Network Open.*

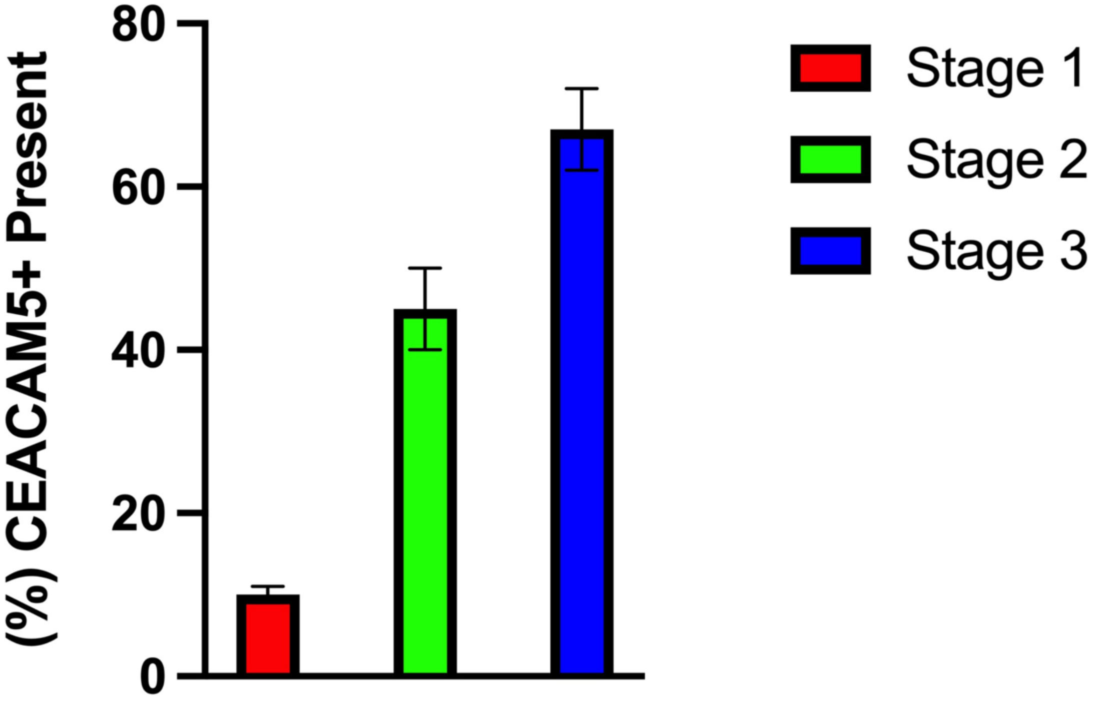

eFigure 2. Higher-Stage Lung Adenocarcinoma Correlated With an Increased Rate of CEACAM5 Expression (AJCC 8th ed)

Error bars represent SEM.

© 2023 Azari F et al. *JAMA Network Open.*

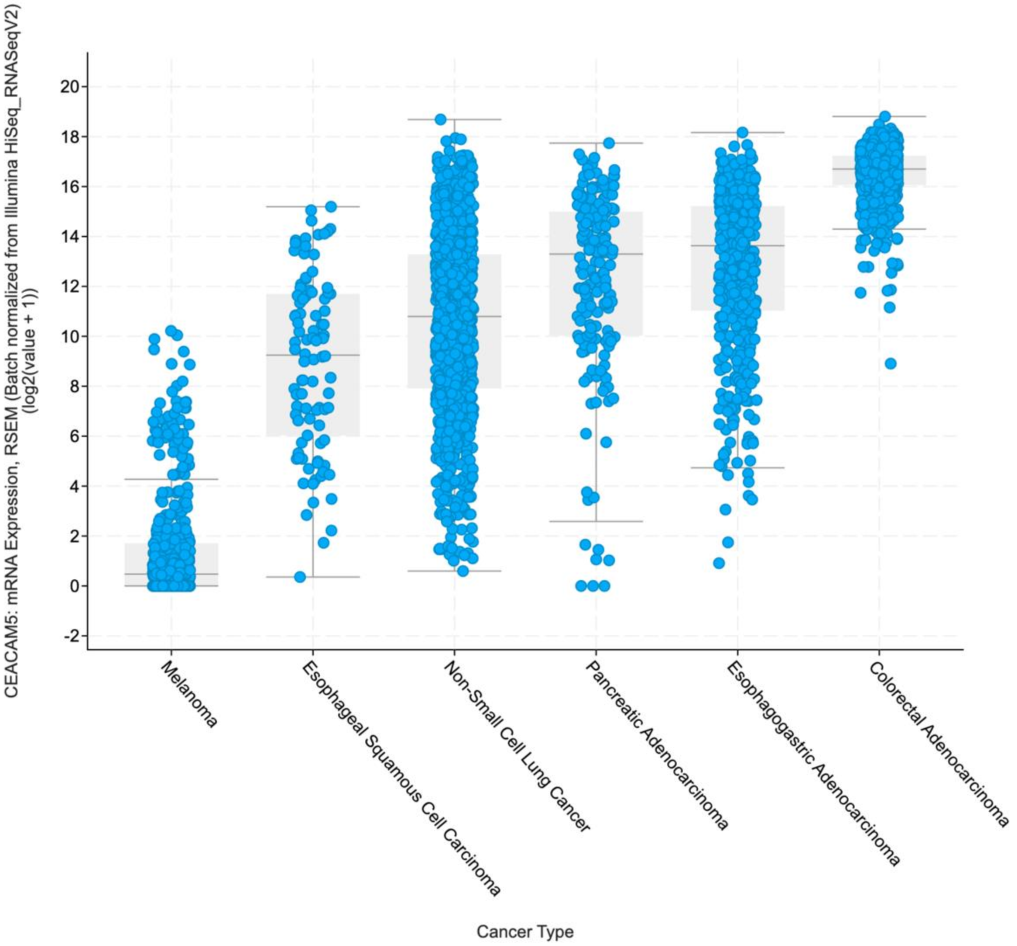

eFigure 3. TCGA Database Analysis Demonstrates CEACAM5 Expression in Various Prevalent Solid Organ Malignancies, With Colorectal Adenocarcinoma Demonstrating the Highest mRNA Expression (16 Log2(Value+1)) and Melanoma Demonstrating the Lowest Median Expression (1 (Log2(Value+1))

NSCLC, esophageal adeno/squamous carcinoma, and pancreatic adenocarcinoma had high CEACAM5 expression of 9.5 (±0.8), and the difference among them was statistically significant (p=0.1).

© 2023 Azari F et al. *JAMA Network Open.*

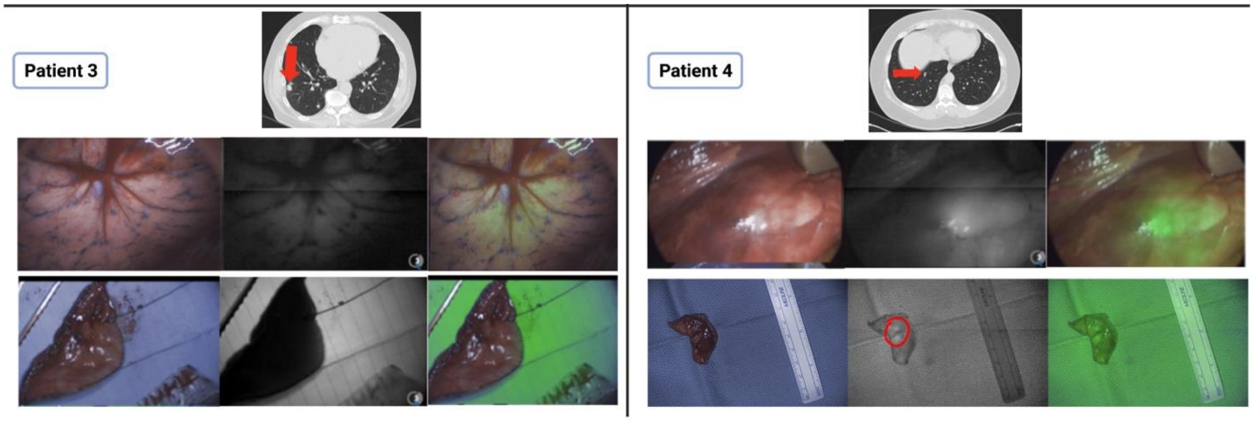

eFigure 4. Patients 3 and 4 With Normal Serum CEA Levels With SGM-101 Ex Vivo Localization of Subcentimeter Nodules

© 2023 Azari F et al. *JAMA Network Open.*

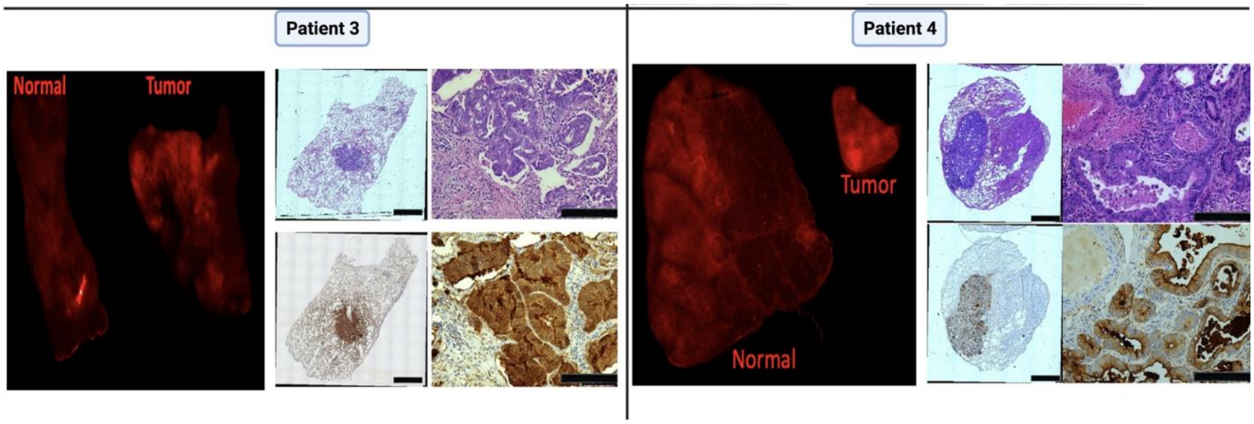

eFigure 5. SGM-101 Fluorescence Correlates With CEACAM5 Presence by IHC (Patient 3) The lesion did not exhibit fluorescence in vivo, the area of tumor foci was small but was detected on the PEARL imager during macroscopic fluorescence assessment. The area of fluorescence was concordant with areas of CEACAM5 expression on IHC. **(Patient 4):** Concordance of SGM-101 fluorescence detected on macroscopic PEARL Imager fluorescence assessment demonstrated small foci of CEACAM5 expression on the tumor concordant with SGM-101 localization. (Scale bar=100 µm).

© 2023 Azari F et al. *JAMA Network Open.*

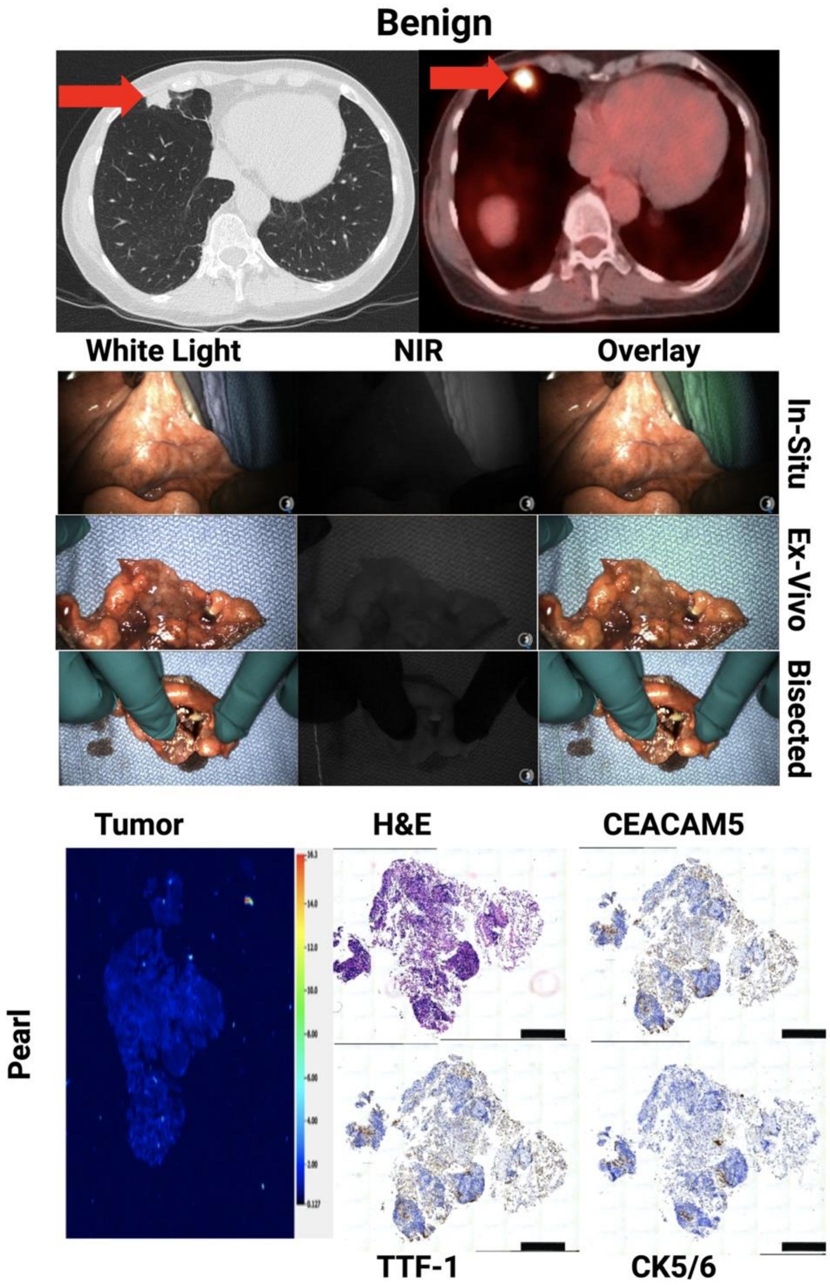

eFigure 6. SGM-101 Localization to CEACAM5+ Areas

Patient with benign lesion finding demonstrating no CEACAM5 staining and SGM-101 fluorescence.

© 2023 Azari F et al. *JAMA Network Open.*

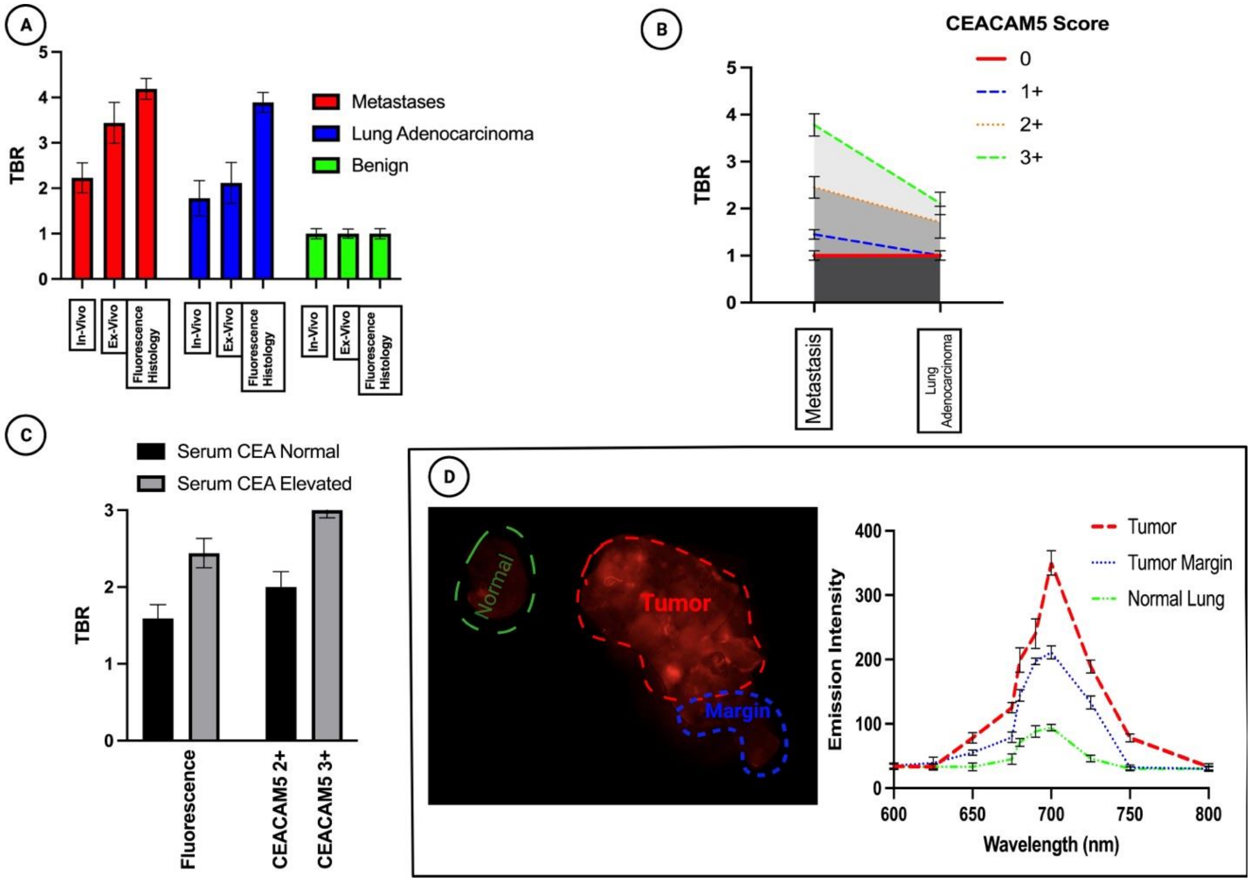

eFigure 7. CEACAM5 IHC and Serum CEACAM5/CEA Can Predict SGM-101 Fluorescence Success and Tumor Localization

(A) Lung nodules can demonstrate excellent in vivo, ex vivo and histologic fluorescence in CEACAM5+ tumors. Ex vivo fluorescence in both cohorts was significantly higher than in vivo fluorescence, likely due to the depth of the lesion, a known limitation of NIR-I technology. **(B)** CEACAM5+ staining score correlates with SGM-101 fluorescence, particularly for primary lung nodules. **(C)** Serum CEA has a positive predictive value for SGM-101 but lacks a negative predictive value**. (D)** CEACAM5+ tumors had the highest fluorescence of SGM-101 compared to margins and normal lungs related to the antigenic concentration of surface glycoproteins.

*Error bars represent SD from triplicate TBR or emission intensity measurements

© 2023 Azari F et al. *JAMA Network Open.*

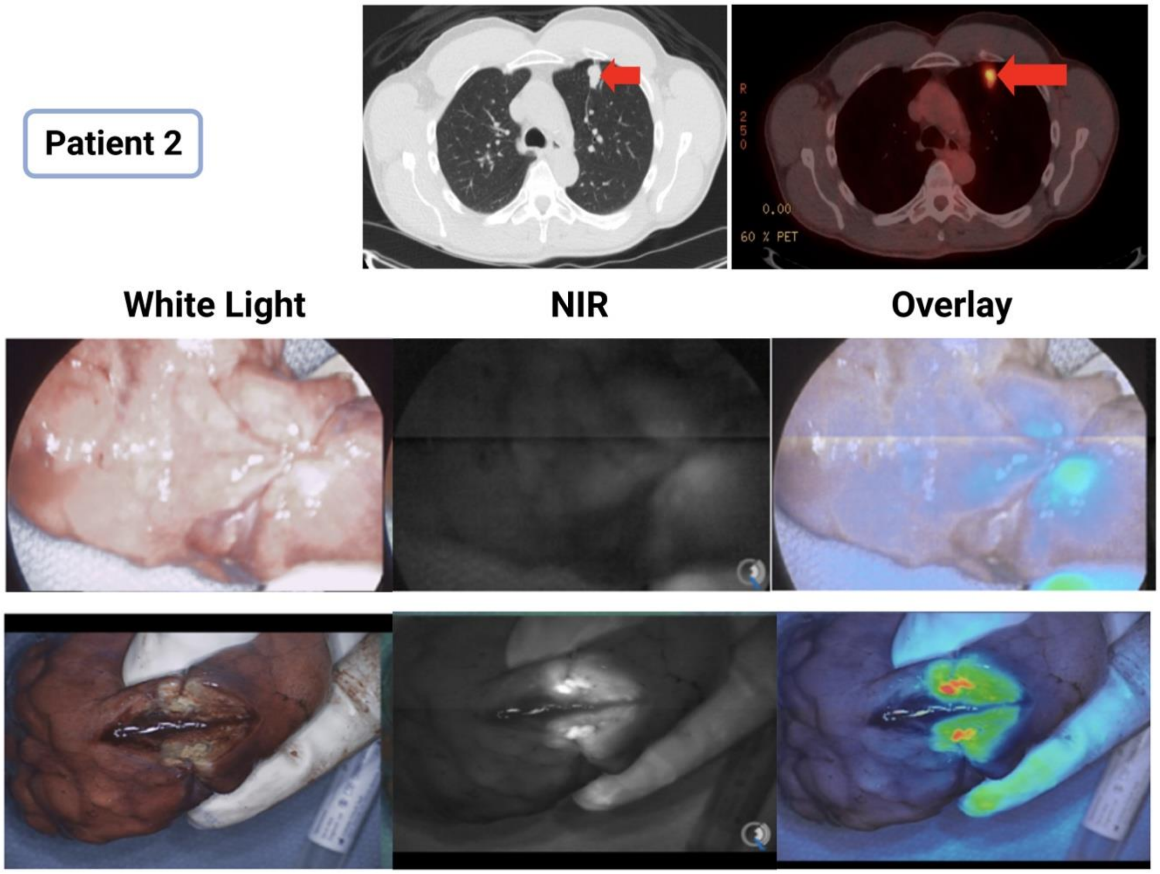

eFigure 8. Intraoperative Findings for **Patient 2** in the Metastatic Control Cohort During SGM-101 Guided Lung Nodule Resection

© 2023 Azari F et al. *JAMA Network Open.*

eFigure 9. Overview of CEACAM5-Targeted Fluorescence Localization of Lung Nodules With SGM-101

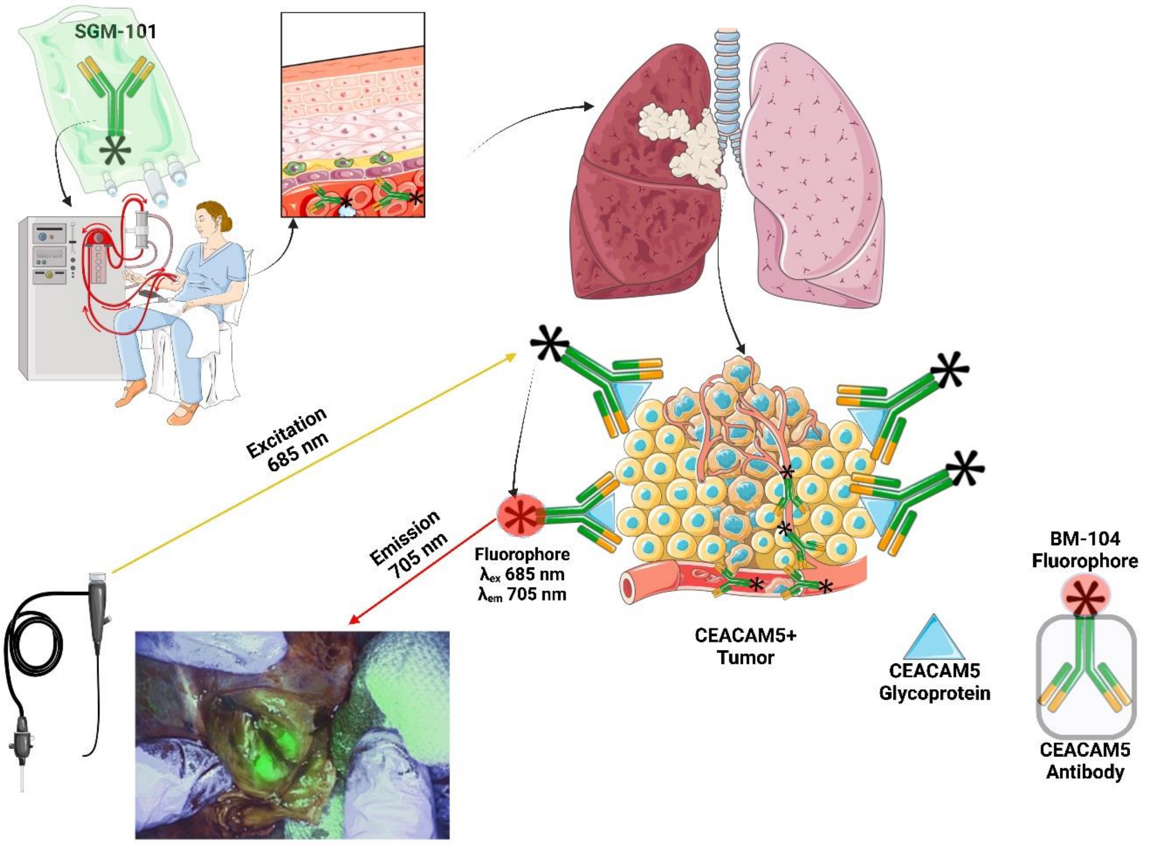

Patients are infused up to 4 days prior to index operation with 10 mg of SMG-101. Subsequently, intraoperative molecular imaging guided lung nodule resection is performed. Fluorescence is predicated upon presence of CEACAM5 glycoprotein on the tumor cell surface due to high antibody-based specificity of the fluorochrome towards CEACAM5.

© 2023 Azari F et al. *JAMA Network Open.*
